# Variation in Soil Respiration across Soil and Vegetation Types in an Alpine Valley

**DOI:** 10.1371/journal.pone.0163968

**Published:** 2016-09-29

**Authors:** Stephanie Grand, Aurélie Rubin, Eric P. Verrecchia, Pascal Vittoz

**Affiliations:** 1 Institute of Earth Surface Dynamics, Faculty of Geosciences and the Environment, University of Lausanne, Lausanne, Switzerland; 2 Department of Ecology and Evolution, Faculty of Biology and Medicine, University of Lausanne, Lausanne, Switzerland; Universidade Federal de Vicosa, BRAZIL

## Abstract

**Background and Aims:**

Soils of mountain regions and their associated plant communities are highly diverse over short spatial scales due to the heterogeneity of geological substrates and highly dynamic geomorphic processes. The consequences of this heterogeneity for biogeochemical transfers, however, remain poorly documented. The objective of this study was to quantify the variability of soil-surface carbon dioxide efflux, known as soil respiration (Rs), across soil and vegetation types in an Alpine valley. To this aim, we measured Rs rates during the peak and late growing season (July-October) in 48 plots located in pastoral areas of a small valley of the Swiss Alps.

**Findings:**

Four herbaceous vegetation types were identified, three corresponding to different stages of primary succession (*Petasition paradoxi* in pioneer conditions, *Seslerion* in more advanced stages and *Poion alpinae* replacing the climactic forests), as well as one (*Rumicion alpinae*) corresponding to eutrophic grasslands in intensively grazed areas. Soils were developed on calcareous alluvial and colluvial fan deposits and were classified into six types including three Fluvisols grades and three Cambisols grades. Plant and soil types had a high level of co-occurrence. The strongest predictor of Rs was soil temperature, yet we detected additional explanatory power of sampling month, showing that temporal variation was not entirely reducible to variations in temperature. Vegetation and soil types were also major determinants of Rs. During the warmest month (August), Rs rates varied by over a factor three between soil and vegetation types, ranging from 2.5 μmol m^-2^ s^-1^ in pioneer environments (*Petasition* on Very Young Fluvisols) to 8.5 μmol m^-2^ s^-1^ in differentiated soils supporting nitrophilous species (*Rumicion* on Calcaric Cambisols).

**Conclusions:**

Overall, this study provides quantitative estimates of spatial and temporal variability in Rs in the mountain environment, and demonstrates that estimations of soil carbon efflux at the watershed scale in complex geomorphic terrain have to account for soil and vegetation heterogeneity.

## Introduction

Soils play a major role in the global carbon (C) cycle [1]. Indeed, soil respiration (Rs) is a major contributor to carbon dioxide (CO_2_) release into the atmosphere, with a CO_2_ efflux estimated at 80 Pg C yr^-1^, representing 10% of the atmospheric C content [2] and nine times the recent annual emission from fossil fuels [3]. Producing rigorous estimates of Rs in different landscapes is thus a crucial issue for forecasts of future atmospheric CO_2_ concentrations.

Soil respiration includes CO_2_ production by heterotrophic soil organisms (e.g., bacteria, fungi, invertebrates), autotrophs (e.g. plant roots) [4] and, to a lesser extent, abiotic degradation of organic matter and carbonate weathering [5]. Like most primarily biological reactions, Rs is highly dependent on temperature and adequate water availability (e.g. [2, 6]) and thus on seasonality, with a maximum respiration rate observed during periods of higher temperatures and intermediate soil moisture content [7].

However, Rs models based solely on climatic variables generally fail to satisfactorily represent fine scale spatial variability [8, 9]. The explanatory power of temperature on Rs rate is particularly limited in landscapes with strong ecological heterogeneity, such as arid to semiarid areas [10] and mountain environments [11]. In the latter case, short-scale spatial variation in Rs has indeed been previously reported (see for instance [12, 13, 14]), but remains largely unattributed [15]. Soil and vegetation types have the potential to explain some of this variability [16–18].

Vegetation cover is known to influence Rs [19], and positive correlations have been observed with root biomass [6, 11], aboveground productivity [20] and species richness [21]. Many of the factors influencing Rs vary with primary succession. Primary productivity and biomass generally increase from colonisation of raw substrate to intermediate successional stages [22], inducing concomitant changes in Rs [23]. The variations of Rs among successional stages located within a restricted area have however rarely been documented, even though initial soil and vegetation development have been shown to result in rapid increases in Rs rates. For instance Guelland et al. [24], working in a glacier forefield chronosequence, reported an efflux multiplied by 17 over a 128-year successional time span.

Soils of mountain environments are highly variable over short spatial scales [25], partly due to the heterogeneity of geological substrates and highly dynamic geomorphic processes [26]. Thus, steep gradients of soil development may exist within the same geo-topographic unit [27]. These pedogenic gradients can reasonably be assumed to generate marked contrasts in soil biogeochemical function, yet field-scale evidence on the interrelation of Rs and soil taxonomical units is scarce. This lack of information restricts our ability to accurately account for biogeochemical fluxes at the landscape scale.

The objective of this study was to quantify the effect of variations in soil type and plant cover on Rs in a valley of the Swiss Alps. We monitored 48 plots distributed in a small valley, spanning six soil types and four different grassland communities corresponding to different stages of soil and vegetation development. Measurements occurred during summer and fall months (from July to October). We hypothesized that (1) soil and vegetation type had a major effect on Rs, with CO_2_ efflux increasing with the degree of soil and vegetation development, and that (2) soil temperature exerted the main temporal control on Rs, so that CO_2_ efflux declined consistently across all plots from summer to fall months.

## Materials and Methods

### Site description

**Fig 1:**
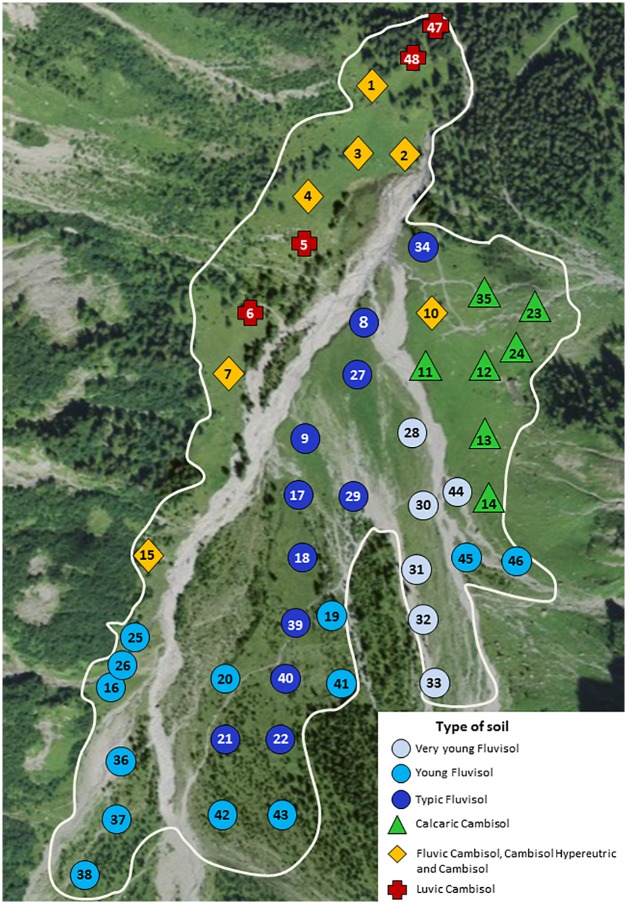
Distribution of soil types among the 48 sampling locations in Vallon de Nant. Rivers are flowing from south to north. Satellite background image reprinted with permission from SwissTopo under a CC BY license, original copyright 2016 swisstopo (BA16073).

The study was conducted in the Nant valley, western Swiss Alps (46°13’N, 07°06’E) at 1500 m above sea level. The Nant valley is a ProNatura protected area and authorization to perform the work was secured through the appropriate authority (Conseil de Coordination Scientifique du Vallon de Nant, University of Lausanne). The study area consisted of a 0.5 km^2^ rangeland under low-intensity grazing by heifers from June to September. Extensive grazing has occurred for centuries and maintains grasslands on stable landscape positions. Forty-eight measurement locations representing different stages of vegetation succession and soil development were selected (Fig 1). Sampling locations were stratified according to vegetation type to ensure representation of each plant community according to its approximate prevalence. Within vegetation type, sampling locations were randomly placed respecting a minimum distance of 50 m between sites. The study was restricted to semi-natural grasslands to avoid the influence of large differences in vegetation biomass between prairie and forested areas.

The mean annual temperature is around 6°C. The coldest month is February with a mean daily minimum of -5°C and a mean daily maximum of 2°C. The hottest month is August with a mean daily minimum of 10°C and a mean daily maximum of 21°C. Cumulative precipitation is around 1800 mm of rain-equivalent evenly distributed throughout the year (values in the same valley at 1250 m asl; [28]). Snow typically covers the ground from November to April and can persist longer along avalanche paths. Solar irradiance is generally weak due to the valley orientation along a north-south axis and steepness of sides and headwall. Soils were formed on old alluvial and colluvial fan deposits [29] with gentle to nearly flat slopes. Floristic inventories, soil classification, and soil respiration measurements were conducted at each location [30].

### Floristic inventories

Exhaustive floristic inventories were performed between July and August 2012 in 4 m^2^ squares. All plant species were identified and ground cover was visually estimated using an 8-grade scale. Hierarchical clustering was used to assign the inventories to vegetation groups. The diagnostic species of each group were selected on the basis of species indicator values [31]. Following these analyses, four groups were retained and each one was matched to a plant alliance according to Delarze et al. [32].

### Soil classification

Auger holes or small profiles were dug at each plots. Each horizon was described in the field in terms of thickness, structure, texture, colour, pH (Hellige pH-Meter, AVM, Freiburg, Germany) and carbonate presence (reaction to HCl 10%). Soils were classified according to the Word Reference Base [33].

### Soil respiration measurements

Measurements of CO_2_ efflux were performed monthly at each plot from July to October 2012 (S1 Table). Sampling days were all chosen during dry weather conditions to avoid potential short pulses of Rs rates due to rain events [5]. Actual dates of the sampling campaigns were July 16 to 19, August 8 to 11, September 17 to 21 and October 17 to 20. All measurements were taken between 10 am and 4 pm. Soil and vegetation types were evenly distributed across sampling times.

Soil respiration was measured both on soil with clipped vegetation (Rsc) and on soil covered by natural vegetation (Rsv). For Rsc, green plants were clipped shortly before measurement at 1.5 cm aboveground using scissors, taking care not to disturb any litter layer. CO_2_ efflux was measured in triplicate on each plot using a portable infrared gas analyser LiCor 8100–103 with a closed dynamic Survey Chamber (LI-COR, Lincoln, Nebraska). The chamber was placed on a 19.9 cm diameter circular collar inserted into the soil at a depth of 0.5 cm shortly prior to measurement. Soil moisture and temperature (at 3 cm depth) were simultaneously recorded using the probes provided with the instrument.

### Statistical analyses of soil respiration

Effects of soil and vegetation on soil CO_2_ efflux were investigated using mixed linear models. There was significant overlap between soil and vegetation types. As a result, two different models were fitted, one with soil and the other with vegetation type as fixed effects. Other fixed terms included sampling month and the interaction between month and soil / vegetation type. Fluxes were standardized to mean daytime monthly temperature to remove effects of temperature drifts between measurements at each plot. Observations were blocked by plot using an R-site random effect with an unstructured covariance matrix to account for repeated sampling. The effects of other continuous variables (species richness, plant cover, soil pH and volumetric water content) on Rs rates were investigated in the whole dataset and within each soil and vegetation group using a similar mixed linear model.

The estimation method was set to restricted (residual) maximum likelihood. Model selection including choice of covariance structure for the random effects was done on the basis of the Bayesian Information Criterion. Approximate normality and goodness of fit were assessed on conditional residual plots. Type III F-tests were used for the significance of fixed effects. These tests were computed using the Satterthwaite adjustment for the denominator degrees of freedom [34], which is intended to produce an accurate F approximation for complex models with many sources of variation. Reported means are least-square (conditional) means ± standard error of the mean (SEM). For significant fixed effects, we carried out comparison of means without adjustment for multiple inferences [35]. The alpha level for significance was set at α = 0.05 for all tests.

Lastly, we performed an analysis of effect size to understand the relative importance of soil / vegetation group, soil temperature and sampling month as predictors of Rs. The squared semi-partial correlation coefficient unbiased estimate, ω^2^, was used as a measure of the proportion of total variation accounted for by each effect.

## Results

### Vegetation

Vegetation covered 55 to 99% of the soil surface. Four vegetation groups corresponding to successional dynamics (S2 Table) were obtained. The first group was attributed to *Petasition paradoxi* and represents a pioneer community growing on young soils found along the most dynamic streams in the Nant valley. Plant cover in this group was low (55–74%, S1 Fig). The characteristic species *Petasites paradoxus* was abundant in each plot. *Anthylis vulneraria* and *Dryas octopetala* were also very common in these calcareous environments. The next group was the *Seslerion* alliance and can be thought of as the second stage in colonization, with a higher plant cover (72–98%) and species richness. *Carex sempervirens* and *Alchemilla conjuncta* were very abundant. *Sesleria caerulea*, typical *Poaceae* of this alliance, was also frequent. The third group corresponded to the *Poion alpinae* alliance and was found in previously forested, stable parts of the landscape. Surface cover in this group was almost complete. Abundant species included *Dactylis glomerata*, *Alchemilla vulgaris*, *Trollius europaeus* and *Plantago media*. The last group was that of *Rumicion alpini* and was found in areas of high livestock pressure also previously under forest. This group included trampling-tolerant species as well as species avoided by grazers. Common species were the nitrophilous *Rumex alpinus* and *Taraxacum officinale*, indicative of high soil nutrient status [36]. Cow tracks were covered by *Poa supina* and *Plantago major*. Overall, floristic richness was maximal in *Seslerion* and minimal in *Rumicion alpini* groups (S1 Fig).

### Soils

Six soil groups were identified, roughly representing different stages of a pedogenic evolution gradient (Table 1). The first group, Very Young Fluvisol, was characterized by a thin C horizon formed on gravelly material. Organic matter accumulation was limited to a discontinuous litter layer. Young Fluvisol was the second soil group and included a thin organo-mineral (A) horizon. Typic Fluvisol, the third identified soil group, had more organic matter incorporated in mineral layers and recorded the development of a B horizon. The fourth group, Calcaric Cambisol, was observed on the eastern riverbank at the foot of a stabilized alluvial/colluvial fan (Fig 1). There was a well-structured B horizon and calcium carbonate was present in the whole profile. The next group comprised deeper soils that were completely devoid of carbonates and included a Fluvic Cambisol, a Hypereutric Cambisol, and Cambisols. These soils are thereafter collectively designated as Cambisols. Finally, the last group included Luvic Cambisols that contained an eluvial horizon (E) and a pedogenic clay accumulation at depth indicative of advanced pedological differentiation [37].

**Table 1:** Average properties of the six soil types as observed in the field (means ± SEM).

Soil type	Horizon	Max horizon depth (cm)	Colour	Texture	Structure	pH	HCl reaction
Very young fluvisol (n = 6)	C	5.8 ± 0.6	Grey	Sandy	Single-grained	8.3 ± 0.1	4
Young fluvisol (n = 13)	A	4.9 ± 0.7	Brown	Silt clay loam	Granular	7.0 ± 0.3	2
	C	15.0 ± 1.0	Grey	Silt clay loam	Weak granular to massive	7.7 ± 0.2	3
Typic fluvisol (n = 11)	A	6.0 ± 1.0	Brown	Silt clay loam	Granular	6.5 ± 0.3	2
	B	13.0 ± 1.2	Brown	Silt clay loam	Blocky	7.6 ± 0.2	3
	C	21.3 ± 1.2	Grey	Silt clay loam	Massive	7.3 ± 0.2	3
Calcaric cambisol (n = 7)	A	6.9 ± 1.8	Brown	Silty clay	Granular	6.8 ± 0.2	2
	B	14.3 ± 5.8	Brown	Silty clay loam	Blocky	7.7 ± 0.3	4
	C	21.1 ± 1.3	Grey	Silty clay	Massive	8.5 ± 0.3	4
Cambisol (n = 7)	A	14.3 ± 3.5	Brown	Silty clay	Granular	5.6 ± 0.2	0
	B	54.3 ± 11.9	Brown	Sitly clay	Blocky	5.8 ± 0.3	0
	C	75.2 ± 8.8	Brown	Silt loam	Weak blocky	6.3 ± 0.5	1
Luvic cambisol (n = 4)	A	12.3 ± 4.9	Brown	Silty clay	Granular	4.9 ± 0.4	0
	E	40.3 ± 21.1	Light brown	Silty clay	Weak blocky	4.8 ± 0.3	0
	B	67.2 ± 9.1	Brown	Clay	Blocky	5.8 ± 0.6	0

pH: field pH obtained by the Hellige colorimetric test. HCl reaction: rated on a scale ranging from 0 to 4, with 0 indicating no effervescence and 4 representing a violent reaction with immediate formation and degassing of large bubbles. Median reaction is reported.

Significant associations between soil and vegetation groups were observed (Table 2). *Petasition* was associated with Very Young Fluvisols and this soil / vegetation association represented the earliest stage of landscape evolution. *Seslerion* was found in better-developed Fluvisols while *Poion* was associated with mature Fluvisols and all Cambisols. *Rumicion* (three occurrences) was restricted to the Cambisols and Calcaric Cambisols that were found in the area of highest grazing pressure.

**Table 2:** Contingency table between soil and vegetation groups. Shaded cells indicate a higher association of a soil and vegetation type than would be expected given an even distribution, as evaluated by a chi-square test of independence.

SOIL		VEGETATION				
		*Petasition*	*Seslerion*	*Poion*	*Rumicion*	Total
**Very young fluvisol**	Count	5	1	0	0	6
	Global %	11	2	0	0	13
**Young fluvisol**	Count	0	12	1	0	13
	Global %	0	25	2	0	28
**Typic fluvisol**	Count	0	7	4	0	11
	Global %	0	15	8	0	23
**Calcaric cambisol**	Count	0	1	4	2	7
	Global %	0	2	9	4	15
**Cambisol**	Count	0	0	6	1	7
	Global %	0	0	12	2	14
**Luvic cambisol**	Count	0	0	4	0	4
	Global %	0	0	7	0	7
**Total**	Count	5	21	19	3	48
	Global %	11	45	38	6	100

### Soil respiration

Two measurements of CO_2_ efflux were carried out, one on clipped (Rsc) and the other on vegetated (Rsv) surfaces. The two flux measurement types were significantly correlated (S2 Fig) but Rsv was higher than Rsc, since the former includes the aboveground autotrophic respiration component. The magnitude of the difference between flux measurement types was maximal in August and minimal in October. In October, the difference between flux measurement types was not significantly different from 0 (p = 0.47), indicating that aboveground autotrophic respiration was no longer an important contributor to the ecosystem C efflux, perhaps due to the onset of vegetative dormancy. There was no detectable effect of soil (p = 0.62) or vegetation group (p = 0.88) on the difference between Rsc and Rsv.

**Fig 2:**
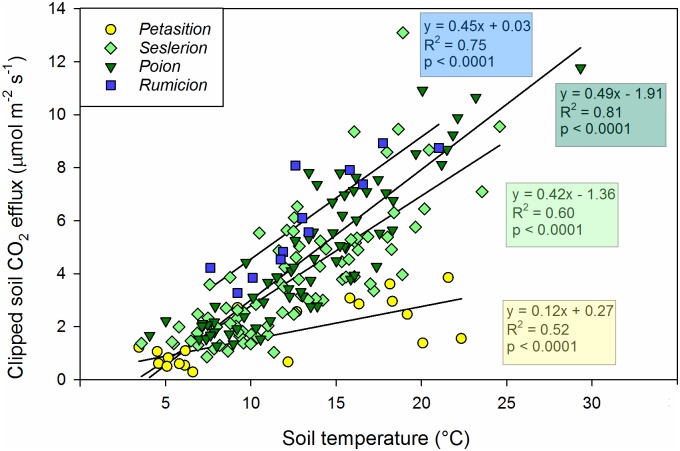
Soil respiration from clipped surfaces in relation to soil temperature measured at 3 cm depth. Results of regression analysis are included for each vegetation type.

Statistical analyses indicated that there were highly significant differences in the magnitude of CO_2_ efflux between soil and vegetation types (S3 and S4 Tables). Other significant effects included measurement month and the interaction between soil / vegetation type and month. The positive dependency of CO_2_ efflux on soil temperature (Fig 2) was also significantly expressed in all models. Plant cover, species richness, soil pH and volumetric water content did not significantly influence Rs rates.

**Fig 3:**
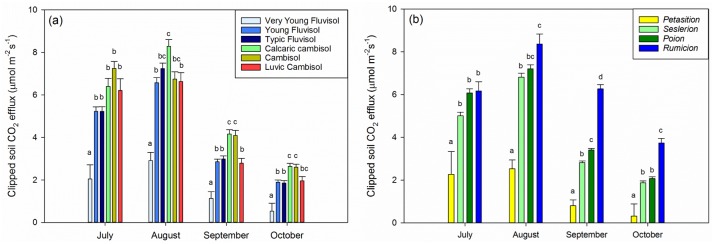
Least square means of clipped soil CO_2_ efflux (μmol m^-2^ s^-1^) across (a) soil and (b) vegetation types from July to October. Within each month, bars topped by a different letter are different at the α = 0.05 level.

**Fig 4:**
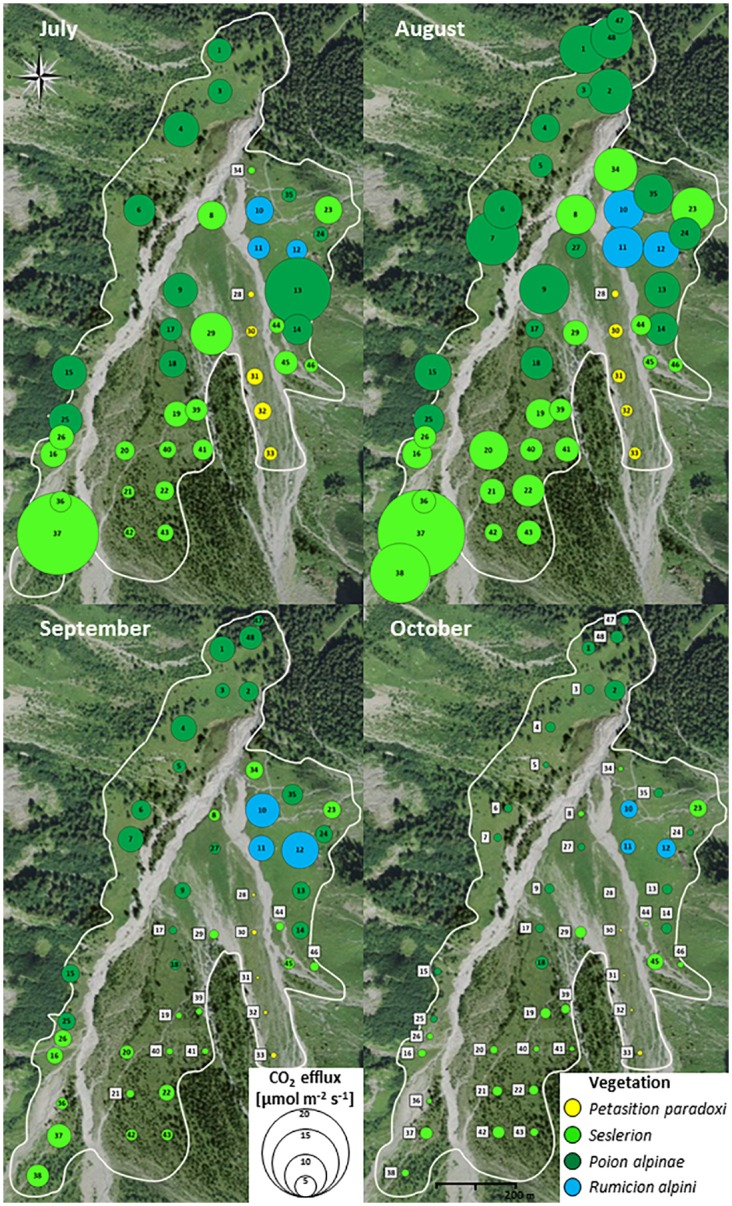
Temporal and spatial variation of CO_2_ efflux (measured on clipped soil surfaces) across vegetation types. Satellite background image reprinted with permission from SwissTopo under a CC BY license, original copyright 2016 swisstopo (BA16073).

For soil groups, Very Young Fluvisols generally had the lowest CO_2_ efflux (1.7 μmol m^-2^ s^-1^ average Rsc), followed by the Young and Typic Fluvisols and the Luvic Cambisols (4.3 μmol m^-2^ s^-1^ average Rsc). Calcaric Cambisols and Cambisols generally had the highest efflux rate (5.3 μmol m^-2^ s^-1^ average Rsc), with the difference reaching statistical significance in September and October (Fig 3). For vegetation, the *Petasition* community generally had the lowest efflux (2.4 μmol m^-2^ s^-1^ average Rsc), followed by *Seslerion* (5.1 μmol m^-2^ s^-1^ average Rsc), *Poion* (5.8 μmol m^-2^ s^-1^ average Rsc) and *Rumicion* (7.3 μmol m^-2^ s^-1^ average Rsc). Differences were most expressed in September (Fig 4).

Effect size analysis showed that models including either soil or vegetation type had very similar explanatory power (Table 3). For each corresponding model, soil and vegetation variables accounted for 16 to 17% of the total variation in Rsc. Soil temperature was, by far, the most important predictor of Rsc, accounting for over 50% of total variance. Sampling month had a significant effect beyond temperature and accounted for an additional 6% of total variation.

**Table 3:** Effect size of explanatory variables (soil or vegetation type, soil temperature and sampling month) on clipped soil respiration. The measure of effect size reported here is the proportion of total variation accounted for (semipartial ω^2^) based on the type I sum of squares.

Soil model	**Overall explanatory power**	**0.74**
	Soil type	0.17
	Soil temperature	0.51
	Sampling month	0.06
Vegetation model	**Overall explanatory power**	**0.74**
	Vegetation type	0.16
	Soil temperature	0.52
	Sampling month	0.06

### Temporal trends in Rs across soil and vegetation groups

Overall, CO_2_ efflux increased by 19% from July to August then decreased by 70% from August to October, as soil temperature dropped (Fig 4). Temporal trends in clipped and vegetated plots showed the same general pattern. The most notable difference was a more pronounced increase in Rsv from July to August. Only trends in Rsc are described below.

**Fig 5:**
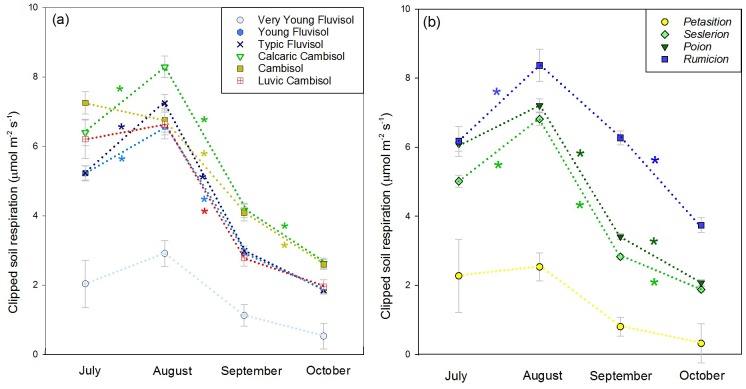
Temporal trends in clipped soil CO_2_ efflux for (a) soil and (b) vegetation types. Stars indicate a statistically significant difference between consecutive months.

In the Very Young Fluvisols and their associated pioneer community (*Petasition*; Table 2), Rsc was low and relatively constant throughout the study period (Fig 5). The Young Fluvisols, Typic Fluvisols, and Luvic Cambisols had higher Rsc and showed very comparable flux values despite the fact that their locations were broadly distributed through the study area (Fig 1). These soils displayed a moderate increase in Rsc (24%) from July to August and a strong decrease from August to September (58%). This temporal trend is also seen in the plant communities associated with these soils, *Seslerion* and *Poion*. The Calcaric Cambisols and Cambisols groups had the highest cumulative Rsc. They were generally found under *Poion*, with occurrences of *Rumicion*. In these soils, fluxes remained significantly higher than in other soils in the fall (S3 Table). This partly reflects the influence of *Rumicion* plots, which showed elevated Rsc in the fall compared to other vegetation groups and a later decline, with the strongest decrease in CO_2_ efflux (40%) occurring between September and October.

## Discussion

### Above and belowground contributions to CO_2_ efflux

In this study, we report CO_2_ efflux from clipped and vegetated surfaces in different soil taxonomic units and plant communities in an Alpine valley. Clipped-soil efflux, Rsc, includes a heterotrophic (soil organic matter decomposition) and an autotrophic (live root respiration) component. On vegetated surfaces, CO_2_ efflux (Rsv) also includes aboveground autotrophic respiration. The photosynthetic offset is expected to be negligible due to the use of dark chambers. The difference between Rsc and Rsv thus represents the aboveground autotrophic contribution. This aboveground contribution did not vary significantly between soil or vegetation types, despite observed differences in species composition and plant cover. Thus, patterns of C allocation were likely similar across plots, perhaps because of the dominance of herbaceous species growing in comparable climatic conditions [38]. This suggests that the main control on autotrophic respiration in this study is climatic and phenologic [39], and that site-dependent factors (soil and vegetation types) are secondary. Differences in CO_2_ efflux detected along the Nant soil and vegetation gradients are thus presumably attributable primarily to differences in heterotrophic respiration.

### Climatic effects on Rs

Soil temperature had a strong positive effect on Rs rates, as expected from global studies [40] and contrary to the report of Geng et al. [41], who proposed that the thermal modulation of Rs in some alpine environments could be unimportant. Within soil and vegetation groups, monthly variability was however not reducible to the effect of temperature. After accounting for temperature differences, we found that Rs rates still increased from July to August then generally decreased in September and October, suggesting an additional control of plant phenology and/or organic substrate availability on Rs.

We estimated the temperature sensitivity of Rs as the slope of the linear relation between Rs and soil temperature. We decided against the use of the widespread Arrhenius or Q10 model [42] because our data showed no evidence of exponential behaviour. The temperature sensitivity of Rs was generally comparable between soil and vegetation groups (Fig 2). A noteworthy exception was for the Very Young Fluvisols under *Petasition*. In this early successional environment, the temperature sensitivity of Rs was almost four times lower than in the other soils (significantly different slope at p < 0.0001). A possible explanation involves substrate limitation, in which the low soil organic matter content keeps Rs low regardless of temperature (see below).

Soil moisture was relatively constant through the study period and was lower for Very Young Fluvisols and Young Fluvisols than for other soils, with maximum divergence observed in July (S3 Fig). Soil moisture had no direct predictive power on Rs rates, nor did it explain any residual variation not accounted for by temperature, either globally or within each soil type. This indicates that contrary to what can be observed in drier environments (e.g. [10, 12]), the dynamics of soil CO_2_ efflux in the valley did not reflect moisture limitation. Moisture content may still be an important determinant of Rs during rain pulses, which were not captured during this study.

### Soil and vegetation effects on Rs

The lower reaches of the Nant valley support, within a small area, soils at an early to intermediate stage of development (Fluvisols and Cambisols) formed on colluvial and alluvial deposits of varying age. This study thus affords the opportunity to compare soils that are representative of the regional mountain environment, within a fairly homogeneous topoclimatic setting [29]. Soils of mountain areas generally warm up slowly after winter, commonly reaching peak temperature in mid-July or a bit later [43]. Because of the high cliffs on the eastern side, the Nant valley receives very limited insolation in spring [29], further delaying the onset of plant growth. In this study extending from July to October, we thus expect to have captured peak annual CO_2_ efflux followed by its fall decline (Fig 5).

We found that soil and vegetation types had a strong influence on CO_2_ efflux. Mean Rsc values in peak season (August) ranged from 2.5 ± 0.8 μmol m^-2^ s^-1^ for poorly developed ecosystems (Very Young Fluvisols under *Petasition*) to 8.4 ± 0.9 μmol m^-2^ s^-1^ for the most eutrophic system (*Rumicion* occurring mostly on Calcaric Cambisols). Moderately developed soils under *Poion* and *Seslerion* showed intermediate Rsc values around 7 ± 0.4 μmol m^-2^ s^-1^. Respiration rates in the Nant Valley’s moderately to well-developed soil groups thus appear to lie in the upper range of reported estimates (4.6–6.5 μmol m^-2^ s^-1^, see [13, 24, 41]), while Rs rates in the youngest edaphic environment were more reminiscent of values reported for high-elevation steppe or tundra conditions (2.0 μmol m^-2^ s^-1^, see [12, 41]). This highlights the need to adequately represent all types of edaphic environments when estimating CO_2_ efflux.

When considering vegetation type, Rs rates increased sequentially with vegetation development. This corroborates and extends the observations of Guelland et al. [24] working on young moraines, to older alpine terrains such as those of the Nant Valley. According to the work of Lane et al. [44] conducted in the same massif, *Petasition* develops on surfaces 5–20 years old and *Seslerion* on surfaces more than 40 years old, while *Poion* and *Rumicion* are believed to occupy surfaces that have been stable for centuries to millennia. Surprisingly, we did not detect any significant effect of plant cover or species richness on Rs, either globally or within each community. This challenges the hypothesis of a universal effect of species richness on soil respiration [21] and instead supports a major role for successional dynamics in determining Rs in grasslands. In *Rumicion* plots which had the lowest species richness, Rs rates were similar to mid- to late-successional communities (*Seslerion* and *Poion*) in summer but declined more slowly in the fall. *Rumicion* plots were located in areas experiencing the highest grazing pressure and dung inputs (heifers are present from July to mid-September). The high Rs rate in late season may reflect a higher nutrient status in these areas, which could work to increase late-season plant productivity and heterotrophic activity.

When considering soil type, the Very Young Fluvisols had the lowest Rs rate, followed by Young Fluvisols, Typic Fluvisols and Luvic Cambisols. Cambisols and Calcaric Cambisols had the highest Rs rate. This sequence is largely consistent with patterns of soil organic matter accrual during soil development. In Very Young Fluvisols, there was no detectable topsoil horizon. Organic C concentration was not measured in this study, but we can conservatively infer from the literature that it was lower than 1% (see for instance [24, 45]). In more advanced stages of pedogenic development, we observed the development of an A horizon with notable accumulation of organics. Soil organic C was measured for a limited number of profiles in the Nant Valley by Gigon [46]. Organic C concentration in the 0–5 cm layer reached 12.6 ± 1.4% for Typic Fluvisols and 13.6 ± 1.2% for Cambisols (S4 Fig). Luvic Cambisols had an organic C depth profile very similar to the Cambisols except for the topmost layer, where C concentrations were lower. This may be due to the onset of eluviation and the leaching of soluble and colloidal organics, and has the potential to explain the lower Rs rate in these soils.

## Conclusions

In this study, we quantified Rs during peak and late growing season in an extensively-managed rangeland of the Swiss Alps and identified its major predictors. Temperature had the largest effect on Rs rates, but did not fully explain temporal trends. The residual monthly variation pointed to substrate limitations or phenologic influence. Soil moisture was not correlated to Rs in this study.

Vegetation and soil types were also important predictors of Rs rates, which generally increased from the *Petasition paradoxi* pioneer communities, to the mid successional *Seslerion* and late successional *Poion alpinae* communities. The *Rumicion alpini* communities found in areas of high nutrient inputs from livestock had the highest Rs rate. Thus Rs increased sequentially along the plant successional and nutrient enrichment gradient. The effect of soil type was somewhat more complex. The least differentiated Very Young Fluvisols had the lowest Rs rate while the more developed Cambisols and Calcaric Cambisols had highest Rs rates. Yet Luvic Cambisols, which can be considered as the most advanced stage of pedogenic development in the valley with the expression of a leached horizon below the organo-mineral layer, showed intermediate Rs rates close to those observed for Young and Typic Fluvisols. This was possibly due to lower organic matter accumulation at the surface. It illustrates the fact that soil biogeochemical function is not subsumed by the conceptual sequence of pedogenic evolution but instead reflects a combination of geochemical, geomorphic, ecological, and management controls.

Overall, this study demonstrates the high spatial heterogeneity of soil CO_2_ efflux at a small scale (~ 0.5 km^2^) and highlights the challenge of producing unbiased regional estimates of Rs in mountain areas. We identified variables (soil and vegetation type), often available from existing mapping data, that can explain some of this variation and thus improve estimates of C fluxes at the watershed scale. The explanatory power of soil and vegetation types was very similar, so that either type of information could be used. In keeping with mounting evidence supporting the inclusion of accurate soil data in global human-Earth system models [47], we propose that a methodology allowing for the assimilation of local, high-resolution soil and vegetation mapping data into soil C models holds the potential for significant improvement in global C cycle estimates.

## Supporting Information

S1 Fig Distribution of ground cover and species richness in the four vegetation types. (TIF)

S2 Fig Relation between CO_2_ efflux measured on clipped and vegetated surfaces. (TIF)

S3 Fig Monthly variation in volumetric water content across soil types. (TIF)

S4 Fig Soil organic carbon depth profile in Typic Fluvisols (n = 2), Cambisols (n = 2) and Luvic Cambisols (n = 2). (TIF)

S1 Table Data matrix for soil respiration measured on clipped (Rsc) and vegetated (Rsv) surfaces. (DOCX)

S2 Table Exhaustive plant inventories for the 48 plots (4 m^2^), classified in groups according to hierarchical clustering. **Species significantly attributed to a group are in the upper part of the table. Species cover: r, a few individuals; +, <1%; 1, 1–5%; 2a, 6–15%; 2b, 16–25%; 3, 26–50%; 4, 51–75%; 5, >75%**. Yellow: plots assigned to Petasition paradoxi; light green: plots assigned to Seslerion; dark green: plots assigned to Poion alpinae; blue: plots assigned to Rumicion alpini. Type of soil: 1, very young Fluvisol; 2, young Fluvisol; 3, typical Fluvisol; 4, Calcaric Cambisol; 5, Cambisol; 6, Luvic Cambisol. Surface area was partitioned between vascular plant cover (herbs), mosses and lichens, bare soil (visible organic or mineral material < 2mm), rocks and pebbles (> 2mm) and litter.
(DOCX)

S3 Table Least square means ± SEM of CO_2_ efflux (μmol m^-2^ s^-1^) across soil types from July to October. For each column and flux measurement type, means followed by a different letter are different at the α = 0.05 level. (a) Clipped soil fluxes (Rsc). (b) Vegetated soil fluxes (Rsv).
(DOCX)

S4 Table Least square means ± SEM of CO_2_ efflux (μmol m^-2^ s^-1^) across vegetation types from July to October. For each column and flux measurement type, means followed by a different letter are different at the α = 0.05 level. (a) Clipped soil fluxes (Rsc). (b) Vegetated soil fluxes (Rsv).
(DOCX)
