# Supplementary figures and images for: Variation in Soil Respiration across Soil and Vegetation Types in an Alpine Valley
Source: PLoS One. 2016 Sep 29;11(9):e0163968. doi: 10.1371/journal.pone.0163968 (PMC5042455; doi:10.1371/journal.pone.0163968)

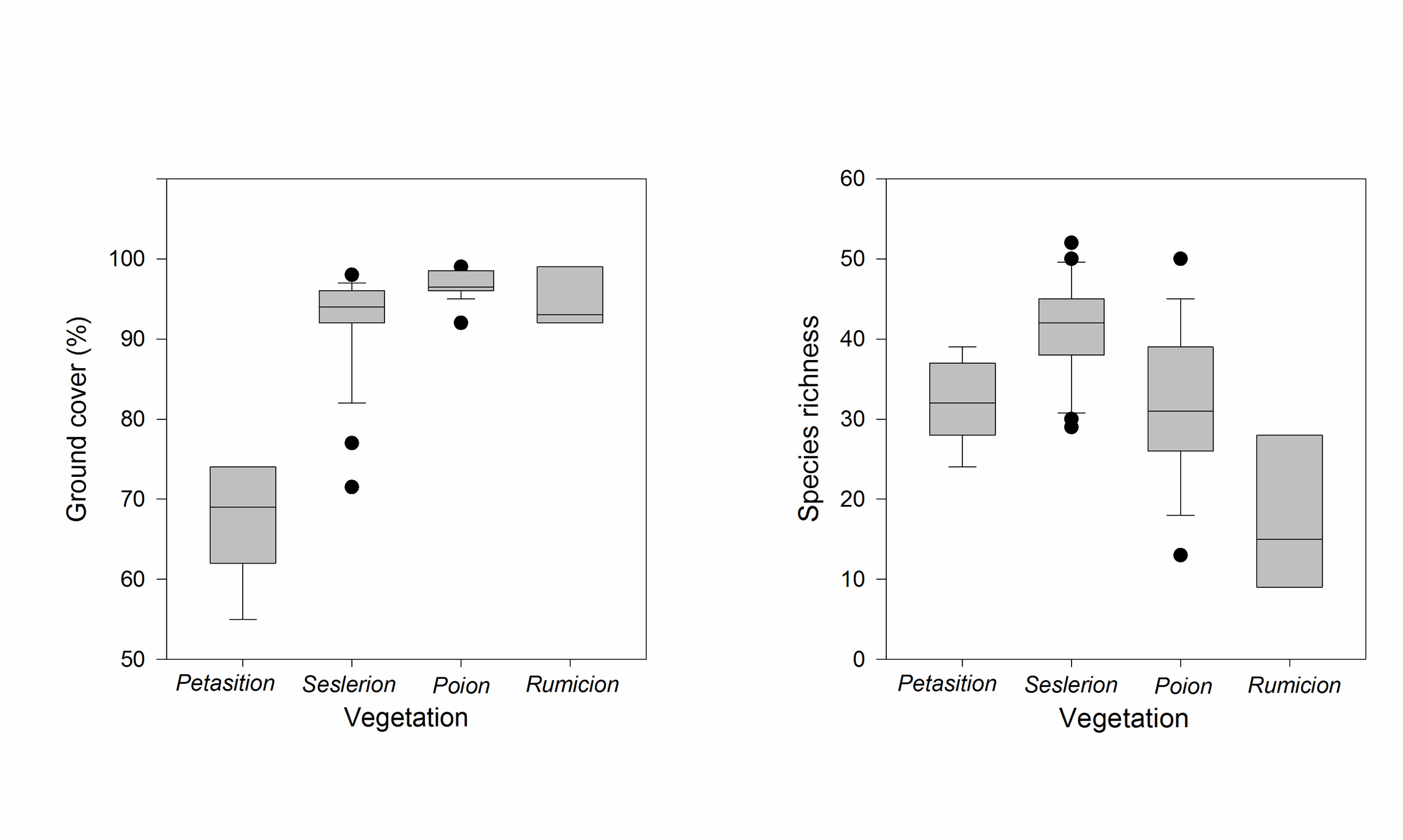

Supplement: S1 Fig — (TIF) [file pone.0163968.s001.tif]

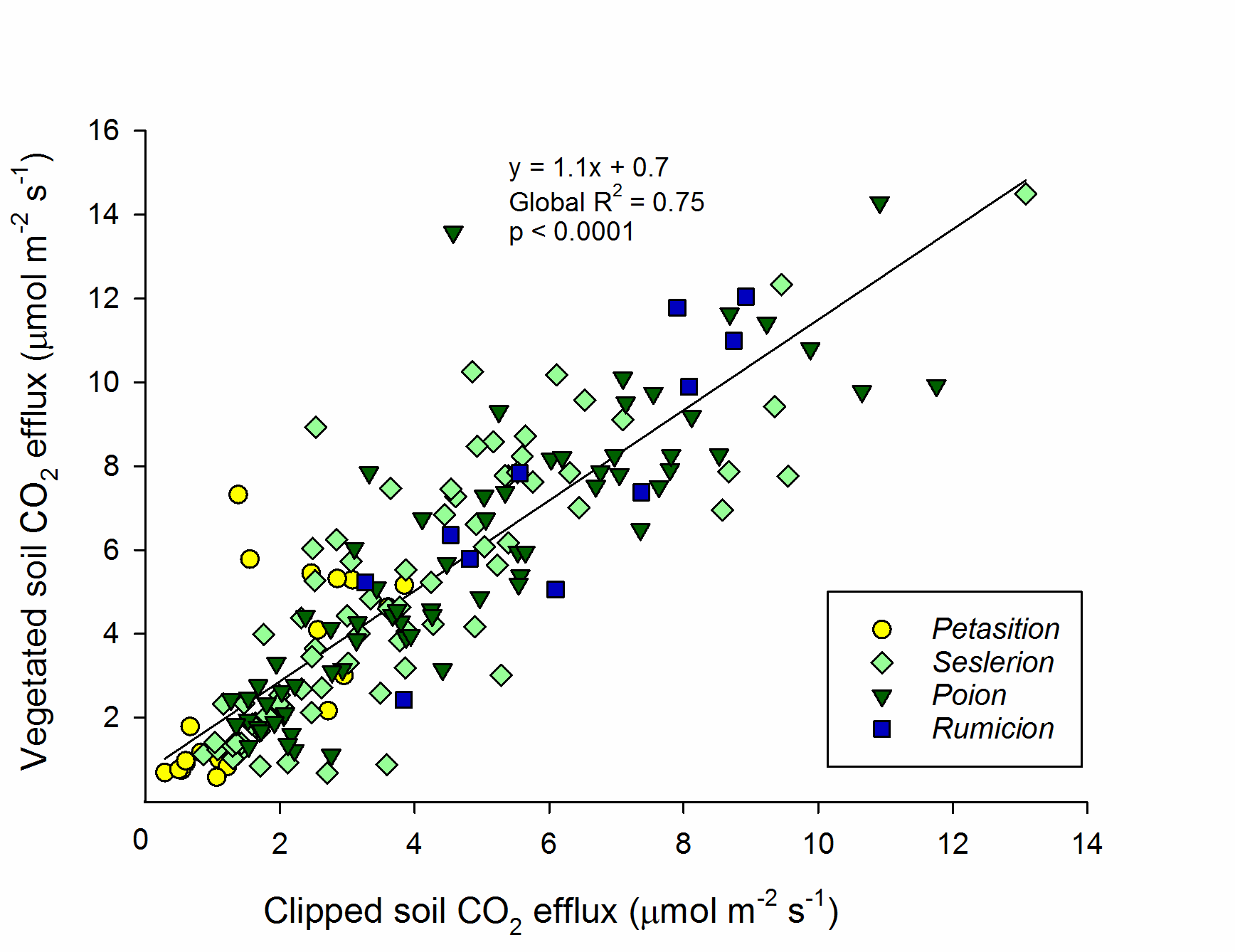

Supplement: S2 Fig — (TIF) [file pone.0163968.s002.tif]

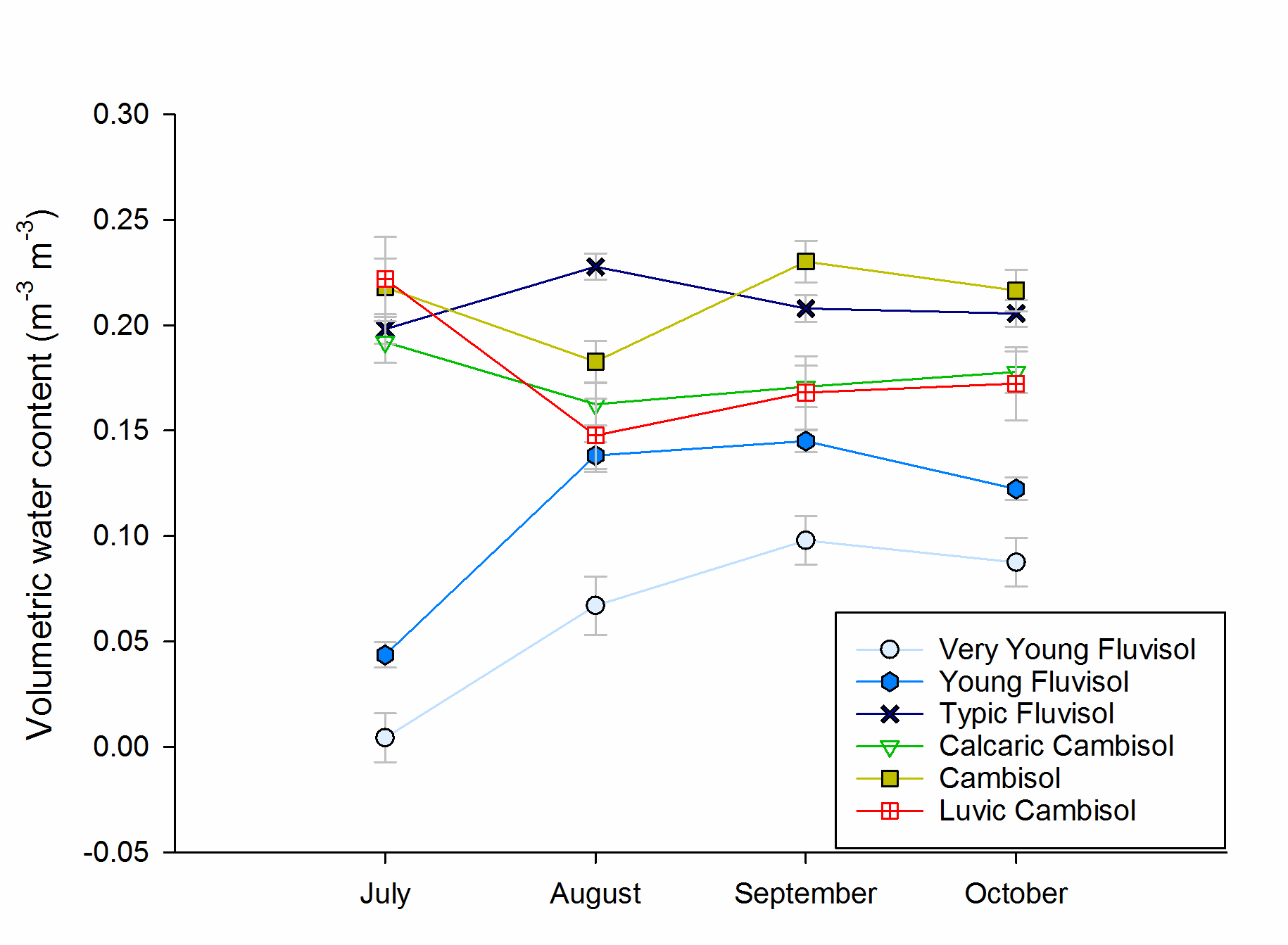

Supplement: S3 Fig — (TIF) [file pone.0163968.s003.tif]

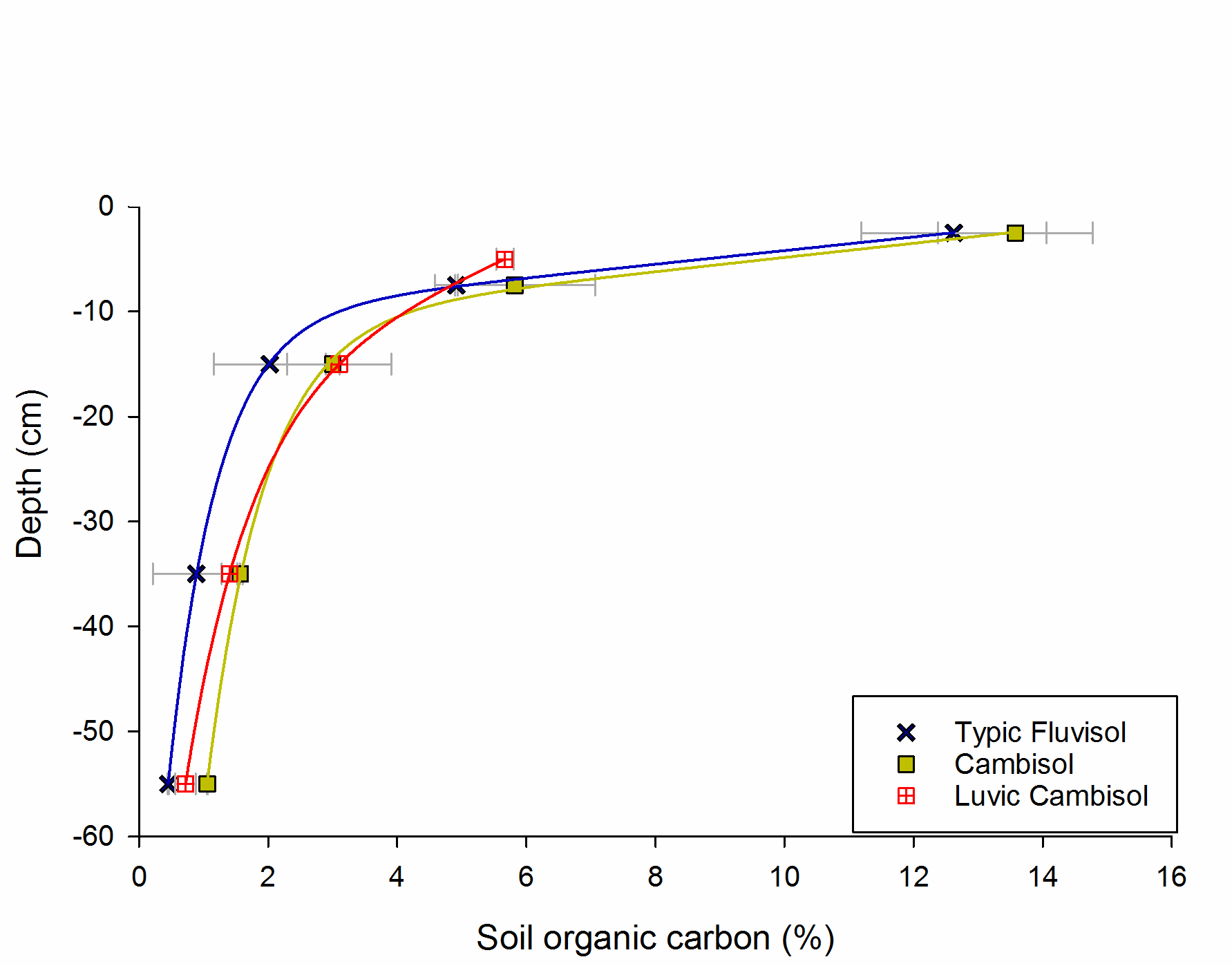

Supplement: S4 Fig — (TIF) [file pone.0163968.s004.tif]
