# Supplementary material for: Variation in Soil Respiration across Soil and Vegetation Types in an Alpine Valley
Source: PLoS One. 2016 Sep 29;11(9):e0163968. doi: 10.1371/journal.pone.0163968 (PMC5042455; doi:10.1371/journal.pone.0163968)
Supplement: S1 Table — (DOCX) [file pone.0163968.s005.docx]

| **Site** | **Xcoord** | **Ycoord** | **Elevation** | **Soil** | **Vegetation** | **Month** | **Rsc** | **Rsv** | **Tchamber** | **Tsoil** | **VWC** |
| --- | --- | --- | --- | --- | --- | --- | --- | --- | --- | --- | --- |
| **ID** |  |  | **m** | **type** | **type** |  | **µmol/m^2^/s** | | **°C** | **°C** | **m^3^/m^3^** |
| **1** | 573938 | 120128 | 1475 | Cambisol | Poion alpinae | 7 | 5.35 | 7.38 | 22.45 | 13.39 | 0.1728 |
| **3** | 573939 | 120045 | 1476 | Cambisol | Poion alpinae | 7 | 5.25 | 9.31 | 23.18 | 12.60 | 0.1728 |
| **4** | 573858 | 119969 | 1492 | Cambisol | Poion alpinae | 7 | 7.81 | 8.26 | 25.53 | 13.39 | 0.1601 |
| **6** | 573777 | 119805 | 1506 | Luvic Cambisol | Poion alpinae | 7 | 6.97 | 8.25 | 25.98 | 15.49 | 0.1719 |
| **8** | 573922 | 119794 | 1485 | Typic Fluvisol | Seslerion | 7 | 6.31 | 7.84 | 22.66 | 18.38 | 0.1474 |
| **9** | 573856 | 119643 | 1500 | Typic Fluvisol | Poion alpinae | 7 | 7.55 | 9.74 | 21.88 | 17.45 | 0.1505 |
| **10** | 574022 | 119804 | 1487 | Cambisol | Rumicion alpini | 7 | 6.10 | 5.05 | 15.72 | 13.00 | 0.1728 |
| **11** | 574020 | 119728 | 1495 | Calcaric Cambisol | Rumicion alpini | 7 | 4.82 | 5.78 | 16.16 | 11.88 | 0.1968 |
| **12** | 574100 | 119724 | 1498 | Calcaric Cambisol | Rumicion alpini | 7 | 4.53 | 6.36 | 15.63 | 11.74 | 0.0753 |
| **13** | 574102 | 119642 | 1508 | Calcaric Cambisol | Poion alpinae | 7 |  | 9.65 | 17.63 | 12.02 | 0.2108 |
| **14** | 574101 | 119563 | 1522 | Calcaric Cambisol | Poion alpinae | 7 | 6.70 | 7.52 | 17.71 | 14.77 | 0.1614 |
| **15** | 573658 | 119476 | 1527 | Cambisol | Poion alpinae | 7 | 7.63 | 7.51 | 24.92 | 16.16 | 0.1610 |
| **16** | 573598 | 119311 | 1545 | Young Fluvisol | Seslerion | 7 | 5.76 | 7.63 | 26.16 | 19.65 | 0.0076 |
| **17** | 573836 | 119563 | 1510 | Typic Fluvisol | Poion alpinae | 7 | 4.97 | 4.87 | 20.36 | 15.56 | 0.1929 |
| **18** | 573840 | 119494 | 1519 | Typic Fluvisol | Poion alpinae | 7 | 6.03 | 8.17 | 20.06 | 16.18 | 0.1789 |
| **19** | 573858 | 119392 | 1536 | Young Fluvisol | Seslerion | 7 | 5.65 | 8.72 | 22.53 | 12.10 | 0.0365 |
| **20** | 573750 | 119318 | 1539 | Young Fluvisol | Seslerion | 7 | 4.25 | 5.23 | 16.28 | 11.98 | 0.0448 |
| **21** | 573757 | 119235 | 1552 | Typic Fluvisol | Seslerion | 7 | 2.84 | 6.25 | 16.62 | 10.30 | 0.1544 |
| **22** | 573840 | 119237 | 1564 | Typic Fluvisol | Seslerion | 7 | 4.28 | 4.22 | 19.20 | 14.04 | 0.1776 |
| **23** | 574166 | 119805 | 1508 | Calcaric Cambisol | Seslerion | 7 | 6.11 | 10.18 | 17.25 | 12.54 | 0.0718 |
| **24** | 574161 | 119753 | 1498 | Calcaric Cambisol | Poion alpinae | 7 | 3.15 | 4.26 | 17.63 | 13.04 | 0.1164 |
| **25** | 573649 | 119380 | 1532 | Young Fluvisol | Poion alpinae | 7 | 7.36 | 6.49 | 26.32 | 13.88 | 0.1151 |
| **26** | 573618 | 119345 | 1539 | Young Fluvisol | Seslerion | 7 | 5.39 | 6.17 | 26.86 | 18.01 | -0.0597 |
| **28** | 574004 | 119634 | 1505 | Very young Fluvisol | Petasition paradoxi | 7 | 1.38 | 7.32 | 22.06 | 20.08 | -0.0929 |
| **29** | 573921 | 119555 | 1513 | Typic Fluvisol | Seslerion | 7 | 9.35 | 9.43 | 20.68 | 16.05 | 0.1208 |
| **30** | 574005 | 119559 | 1516 | Very young Fluvisol | Petasition paradoxi | 7 | 2.46 | 5.44 | 20.77 | 19.17 | -0.0518 |
| **31** | 574012 | 119468 | 1525 | Very young Fluvisol | Petasition paradoxi | 7 | 3.61 | 4.63 | 23.68 | 18.16 | -0.0269 |
| **32** | 574013 | 119398 | 1540 | Very young Fluvisol | Petasition paradoxi | 7 | 3.85 | 5.16 | 21.38 | 21.57 | -0.1257 |
| **33** | 574030 | 119312 | 1560 | Very young Fluvisol | Petasition paradoxi | 7 | 2.95 | 3.00 | 21.36 | 18.29 | 0.0028 |
| **34** | 574007 | 119828 | 1480 | Typic Fluvisol | Seslerion | 7 | 1.59 | 1.84 | 14.94 | 10.38 | -0.0007 |
| **35** | 574084 | 119836 | 1495 | Calcaric Cambisol | Poion alpinae | 7 | 3.14 | 3.85 | 15.50 | 12.27 | 0.1610 |
| **36** | 573631 | 119215 | 1551 | Young Fluvisol | Seslerion | 7 | 4.61 | 7.27 | 23.36 | 12.64 | 0.0006 |
| **37** | 573599 | 119150 | 1562 | Young Fluvisol | Seslerion | 7 |  | 6.80 | 26.94 | 19.16 | 0.0670 |
| **39** | 573900 | 119400 | 1537 | Typic Fluvisol | Seslerion | 7 | 4.86 | 10.26 | 25.12 | 11.64 | 0.1566 |
| **40** | 573839 | 119320 | 1549 | Typic Fluvisol | Seslerion | 7 | 3.78 | 3.83 | 17.91 | 12.80 | 0.2038 |
| **41** | 573913 | 119320 | 1557 | Young Fluvisol | Seslerion | 7 | 4.54 | 7.45 | 21.66 | 15.75 | -0.0392 |
| **42** | 573760 | 119152 | 1561 | Young Fluvisol | Seslerion | 7 | 2.53 | 8.93 | 17.26 | 11.85 | -0.2974 |
| **43** | 573834 | 119152 | 1578 | Young Fluvisol | Seslerion | 7 | 3.65 | 7.47 | 17.92 | 11.72 | 0.1059 |
| **44** | 574058 | 119571 | 1516 | Very young Fluvisol | Seslerion | 7 | 3.35 | 4.84 | 17.25 | 17.20 | 0.0203 |
| **45** | 574077 | 119463 | 1530 | Young Fluvisol | Seslerion | 7 | 5.23 | 5.63 | 20.82 | 14.06 | -0.0558 |
| **46** | 574125 | 119480 | 1538 | Young Fluvisol | Seslerion | 7 | 3.00 | 4.43 | 20.96 | 14.22 | -0.0029 |
| **1** | 573938 | 120128 | 1475 | Cambisol | Poion alpinae | 8 | 10.65 | 9.78 | 23.10 | 23.19 | 0.1745 |
| **2** | 573993 | 120043 | 1474 | Cambisol | Poion alpinae | 8 | 9.88 | 10.81 | 25.39 | 22.12 | 0.1282 |
| **3** | 573939 | 120045 | 1476 | Cambisol | Poion alpinae | 8 | 3.32 | 7.84 | 16.98 | 12.93 | 0.2073 |
| **4** | 573858 | 119969 | 1492 | Cambisol | Poion alpinae | 8 | 6.20 | 8.21 | 20.80 | 15.36 | 0.1793 |
| **5** | 573849 | 119893 | 1491 | Luvic Cambisol | Poion alpinae | 8 | 5.03 | 7.29 | 22.54 | 15.49 | -0.0518 |
| **6** | 573777 | 119805 | 1506 | Luvic Cambisol | Poion alpinae | 8 | 8.53 | 8.27 | 23.88 | 19.67 | 0.1405 |
| **7** | 573748 | 119746 | 1498 | Cambisol | Poion alpinae | 8 | 11.76 | 9.93 | 23.15 | 29.32 | -0.1182 |
| **8** | 573922 | 119794 | 1485 | Typic Fluvisol | Seslerion | 8 | 8.67 | 7.87 | 23.15 | 20.45 | 0.1951 |
| **9** | 573856 | 119643 | 1500 | Typic Fluvisol | Poion alpinae | 8 | 10.91 | 14.29 | 23.71 | 20.06 | 0.1807 |
| **10** | 574022 | 119804 | 1487 | Cambisol | Rumicion alpini | 8 | 8.74 | 10.99 | 22.26 | 21.02 | 0.1968 |
| **11** | 574020 | 119728 | 1495 | Calcaric Cambisol | Rumicion alpini | 8 | 8.92 | 12.05 | 22.71 | 17.74 | 0.1544 |
| **12** | 574100 | 119724 | 1498 | Calcaric Cambisol | Rumicion alpini | 8 | 7.91 | 11.78 | 21.29 | 15.79 |  |
| **13** | 574102 | 119642 | 1508 | Calcaric Cambisol | Poion alpinae | 8 | 7.80 | 7.92 | 20.40 | 15.21 |  |
| **14** | 574101 | 119563 | 1522 | Calcaric Cambisol | Poion alpinae | 8 | 7.14 | 9.51 | 21.63 | 16.02 |  |
| **15** | 573658 | 119476 | 1527 | Cambisol | Poion alpinae | 8 | 8.12 | 9.19 | 23.31 | 21.18 | 0.1610 |
| **16** | 573598 | 119311 | 1545 | Young Fluvisol | Seslerion | 8 | 6.45 | 7.01 | 23.26 | 20.19 | 0.1383 |
| **17** | 573836 | 119563 | 1510 | Typic Fluvisol | Poion alpinae | 8 | 4.11 | 6.75 | 20.83 | 12.36 | 0.2021 |
| **18** | 573840 | 119494 | 1519 | Typic Fluvisol | Poion alpinae | 8 | 7.10 | 10.10 | 22.41 | 16.78 | 0.1946 |
| **19** | 573858 | 119392 | 1536 | Young Fluvisol | Seslerion | 8 | 6.53 | 9.58 | 22.21 | 12.72 | 0.2104 |
| **20** | 573750 | 119318 | 1539 | Young Fluvisol | Seslerion | 8 | 8.58 | 6.95 | 22.97 | 17.97 | 0.0850 |
| **21** | 573757 | 119235 | 1552 | Typic Fluvisol | Seslerion | 8 | 5.60 | 8.23 | 20.96 | 12.49 | 0.1807 |
| **22** | 573840 | 119237 | 1564 | Typic Fluvisol | Seslerion | 8 | 7.10 | 9.11 | 24.19 | 23.55 | 0.1859 |
| **23** | 574166 | 119805 | 1508 | Calcaric Cambisol | Seslerion | 8 | 9.45 | 12.33 | 24.07 | 18.65 | 0.0207 |
| **24** | 574161 | 119753 | 1498 | Calcaric Cambisol | Poion alpinae | 8 | 7.05 | 7.81 | 22.63 | 17.92 |  |
| **25** | 573649 | 119380 | 1532 | Young Fluvisol | Poion alpinae | 8 | 6.77 | 7.87 | 24.92 | 18.34 | 0.1422 |
| **26** | 573618 | 119345 | 1539 | Young Fluvisol | Seslerion | 8 | 4.91 | 6.61 | 23.11 | 18.24 | -0.1113 |
| **27** | 573923 | 119727 | 1494 | Typic Fluvisol | Poion alpinae | 8 | 4.58 | 13.58 | 23.27 | 13.71 | 0.1627 |
| **28** | 574004 | 119634 | 1505 | Very young Fluvisol | Petasition paradoxi | 8 | 1.55 | 5.77 | 24.69 | 22.34 | -0.0475 |
| **29** | 573921 | 119555 | 1513 | Typic Fluvisol | Seslerion | 8 | 5.53 | 7.85 | 15.77 | 10.49 | 0.2204 |
| **30** | 574005 | 119559 | 1516 | Very young Fluvisol | Petasition paradoxi | 8 | 3.07 | 5.28 | 23.60 | 15.80 | 0.0203 |
| **31** | 574012 | 119468 | 1525 | Very young Fluvisol | Petasition paradoxi | 8 | 2.85 | 5.31 | 20.46 | 16.34 | 0.0351 |
| **32** | 574013 | 119398 | 1540 | Very young Fluvisol | Petasition paradoxi | 8 | 2.56 | 4.09 | 15.23 | 12.69 | -0.0024 |
| **33** | 574030 | 119312 | 100 | Very young Fluvisol | Petasition paradoxi | 8 | 2.72 | 2.16 | 8.86 | 9.21 | 0.0793 |
| **34** | 574007 | 119828 | 1480 | Typic Fluvisol | Seslerion | 8 | 9.55 | 7.76 | 23.76 | 24.59 | 0.0649 |
| **35** | 574084 | 119836 | 1495 | Calcaric Cambisol | Poion alpinae | 8 | 8.69 | 11.63 | 24.00 | 21.50 | 0.1623 |
| **36** | 573631 | 119215 | 1551 | Young Fluvisol | Seslerion | 8 | 5.17 | 8.58 | 25.28 | 16.13 | 0.1610 |
| **37** | 573599 | 119150 | 1562 | Young Fluvisol | Seslerion | 8 |  | 8.65 | 26.45 | 22.86 | 0.1391 |
| **38** | 573604 | 119058 | 1576 | Young Fluvisol | Seslerion | 8 | 13.09 | 14.49 | 25.41 | 18.92 | 0.1142 |
| **39** | 573900 | 119400 | 1537 | Typic Fluvisol | Seslerion | 8 | 4.93 | 8.48 | 24.00 | 14.39 | 0.1846 |
| **40** | 573839 | 119320 | 1549 | Typic Fluvisol | Seslerion | 8 | 5.04 | 6.07 | 19.56 | 12.84 | 0.1837 |
| **41** | 573913 | 119320 | 1557 | Young Fluvisol | Seslerion | 8 | 5.35 | 7.78 | 25.59 | 16.21 | 0.0740 |
| **42** | 573760 | 119152 | 1561 | Young Fluvisol | Seslerion | 8 | 3.97 |  | 24.05 | 18.88 | -0.0142 |
| **43** | 573834 | 119152 | 1578 | Young Fluvisol | Seslerion | 8 | 5.40 | 7.77 | 26.23 | 16.86 | 0.0321 |
| **44** | 574058 | 119571 | 1516 | Very young Fluvisol | Seslerion | 8 | 4.44 | 6.84 | 20.58 | 15.33 |  |
| **45** | 574077 | 119463 | 1530 | Young Fluvisol | Seslerion | 8 | 3.06 | 5.73 | 18.24 | 13.79 |  |
| **46** | 574125 | 119480 | 1538 | Young Fluvisol | Seslerion | 8 | 3.18 | 4.01 | 16.96 | 12.96 |  |
| **47** | 574014 | 120185 | 1473 | Luvic Cambisol | Poion alpinae | 8 | 5.65 | 5.95 | 21.91 | 18.30 | 0.1885 |
| **48** | 573997 | 120151 | 1473 | Luvic Cambisol | Poion alpinae | 8 | 9.24 | 11.41 | 24.77 | 21.85 | 0.1138 |
| **1** | 573938 | 120128 | 1475 | Cambisol | Poion alpinae | 9 | 5.55 | 5.19 | 22.19 | 14.81 | 0.1828 |
| **2** | 573993 | 120043 | 1474 | Cambisol | Poion alpinae | 9 | 4.25 | 4.57 | 22.61 | 12.51 | 0.1798 |
| **3** | 573939 | 120045 | 1476 | Cambisol | Poion alpinae | 9 | 3.11 | 6.04 | 9.91 | 10.13 | 0.2209 |
| **4** | 573858 | 119969 | 1492 | Cambisol | Poion alpinae | 9 | 5.57 | 5.38 | 18.06 | 13.66 | 0.1724 |
| **5** | 573849 | 119893 | 1491 | Luvic Cambisol | Poion alpinae | 9 | 2.77 | 3.09 | 18.82 | 13.89 | -0.0435 |
| **6** | 573777 | 119805 | 1506 | Luvic Cambisol | Poion alpinae | 9 | 4.26 | 4.44 | 20.71 | 17.36 | 0.1125 |
| **7** | 573748 | 119746 | 1498 | Cambisol | Poion alpinae | 9 | 5.53 | 5.96 | 20.65 | 17.55 | 0.1881 |
| **8** | 573922 | 119794 | 1485 | Typic Fluvisol | Seslerion | 9 | 2.47 | 2.12 | 22.10 | 12.51 | 0.1453 |
| **9** | 573856 | 119643 | 1500 | Typic Fluvisol | Poion alpinae | 9 | 3.43 | 5.09 | 20.85 | 12.21 | 0.2003 |
| **10** | 574022 | 119804 | 1487 | Cambisol | Rumicion alpini | 9 | 7.37 | 7.38 | 19.16 | 16.57 | 0.1413 |
| **11** | 574020 | 119728 | 1495 | Calcaric Cambisol | Rumicion alpini | 9 | 5.56 | 7.83 | 19.20 | 13.38 | 0.1671 |
| **12** | 574100 | 119724 | 1498 | Calcaric Cambisol | Rumicion alpini | 9 | 8.08 | 9.90 | 19.20 | 12.60 | 0.1925 |
| **13** | 574102 | 119642 | 1508 | Calcaric Cambisol | Poion alpinae | 9 | 3.87 | 3.91 | 17.39 | 11.52 | 0.1544 |
| **14** | 574101 | 119563 | 1522 | Calcaric Cambisol | Poion alpinae | 9 | 3.67 | 4.44 | 16.43 | 10.99 | 0.1518 |
| **15** | 573658 | 119476 | 1527 | Cambisol | Poion alpinae | 9 | 3.94 | 3.96 | 21.42 | 16.07 | 0.1759 |
| **16** | 573598 | 119311 | 1545 | Young Fluvisol | Seslerion | 9 | 3.61 | 4.59 | 19.55 | 17.00 | 0.1405 |
| **17** | 573836 | 119563 | 1510 | Typic Fluvisol | Poion alpinae | 9 | 1.67 | 1.78 | 0.29 | 4.07 | 0.2139 |
| **18** | 573840 | 119494 | 1519 | Typic Fluvisol | Poion alpinae | 9 | 2.21 | 1.22 | 0.27 | 5.01 | 0.1636 |
| **19** | 573858 | 119392 | 1536 | Young Fluvisol | Seslerion | 9 | 1.36 | 1.44 | 1.15 | 5.48 | 0.2008 |
| **20** | 573750 | 119318 | 1539 | Young Fluvisol | Seslerion | 9 | 3.01 | 3.30 | 16.46 | 13.36 | 0.0797 |
| **21** | 573757 | 119235 | 1552 | Typic Fluvisol | Seslerion | 9 | 1.76 | 3.98 | 13.39 | 6.85 | 0.1164 |
| **22** | 573840 | 119237 | 1564 | Typic Fluvisol | Seslerion | 9 | 3.49 | 2.58 | 17.09 | 11.93 | 0.1501 |
| **23** | 574166 | 119805 | 1508 | Calcaric Cambisol | Seslerion | 9 | 3.87 | 5.52 | 21.37 | 15.57 | -0.0129 |
| **24** | 574161 | 119753 | 1498 | Calcaric Cambisol | Poion alpinae | 9 | 3.74 | 4.55 | 18.97 | 13.51 | 0.1225 |
| **25** | 573649 | 119380 | 1532 | Young Fluvisol | Poion alpinae | 9 | 3.79 | 4.29 | 24.40 | 15.85 | 0.1431 |
| **26** | 573618 | 119345 | 1539 | Young Fluvisol | Seslerion | 9 | 3.78 | 4.63 | 21.01 | 15.37 | -0.0094 |
| **27** | 573923 | 119727 | 1494 | Typic Fluvisol | Poion alpinae | 9 | 2.38 | 4.42 | 19.06 | 9.60 | 0.1977 |
| **28** | 574004 | 119634 | 1505 | Very young Fluvisol | Petasition paradoxi | 9 | 0.67 | 1.78 | 15.68 | 12.18 | 0.0067 |
| **29** | 573921 | 119555 | 1513 | Typic Fluvisol | Seslerion | 9 | 1.99 | 2.54 | 0.34 | 5.78 | 0.1868 |
| **30** | 574005 | 119559 | 1516 | Very young Fluvisol | Petasition paradoxi | 9 | 1.09 | 1.00 | 10.02 | 6.16 | -0.0011 |
| **31** | 574012 | 119468 | 1525 | Very young Fluvisol | Petasition paradoxi | 9 | 0.61 | 0.91 | 6.03 | 4.59 | 0.1689 |
| **32** | 574013 | 119398 | 1540 | Very young Fluvisol | Petasition paradoxi | 9 | 0.82 | 1.16 | 5.76 | 5.15 | 0.0286 |
| **33** | 574030 | 119312 | 1560 | Very young Fluvisol | Petasition paradoxi | 9 | 1.22 | 0.83 | 5.43 | 3.44 | 0.0408 |
| **34** | 574007 | 119828 | 1480 | Typic Fluvisol | Seslerion | 9 | 3.89 | 4.06 | 19.31 | 16.06 | 0.0509 |
| **35** | 574084 | 119836 | 1495 | Calcaric Cambisol | Poion alpinae | 9 | 4.48 | 5.68 | 20.41 | 15.01 | 0.0705 |
| **36** | 573631 | 119215 | 1551 | Young Fluvisol | Seslerion | 9 | 2.52 | 5.26 | 17.32 | 12.59 | 0.1540 |
| **37** | 573599 | 119150 | 1562 | Young Fluvisol | Seslerion | 9 | 5.28 | 3.01 | 17.53 | 15.86 | 0.1352 |
| **38** | 573604 | 119058 | 1576 | Young Fluvisol | Seslerion | 9 | 4.89 | 4.17 | 21.03 | 16.28 | 0.0710 |
| **39** | 573900 | 119400 | 1537 | Typic Fluvisol | Seslerion | 9 | 1.47 | 2.33 | 9.91 | 6.49 | 0.1815 |
| **40** | 573839 | 119320 | 1549 | Typic Fluvisol | Seslerion | 9 | 1.42 | 1.38 | 8.85 | 5.38 | 0.1300 |
| **41** | 573913 | 119320 | 1557 | Young Fluvisol | Seslerion | 9 | 1.37 | 1.17 | 6.10 | 3.59 | 0.0933 |
| **42** | 573760 | 119152 | 1561 | Young Fluvisol | Seslerion | 9 | 2.48 | 6.04 | 10.90 | 9.45 | 0.0644 |
| **43** | 573834 | 119152 | 1578 | Young Fluvisol | Seslerion | 9 | 2.52 | 3.65 | 16.53 | 9.62 | 0.1558 |
| **44** | 574058 | 119571 | 1516 | Very young Fluvisol | Seslerion | 9 | 1.82 | 2.06 | 13.33 | 9.52 | 0.0434 |
| **45** | 574077 | 119463 | 1530 | Young Fluvisol | Seslerion | 9 | 2.32 | 4.38 | 12.89 | 9.19 | -0.0190 |
| **46** | 574125 | 119480 | 1538 | Young Fluvisol | Seslerion | 9 | 2.02 | 2.20 | 12.41 | 9.14 | 0.0264 |
| **47** | 574014 | 120185 | 1473 | Luvic Cambisol | Poion alpinae | 9 | 2.22 | 2.78 | 20.04 | 11.13 | 0.1916 |
| **48** | 573997 | 120151 | 1473 | Luvic Cambisol | Poion alpinae | 9 | 5.06 | 6.74 | 22.06 | 15.22 | 0.2113 |
| **1** | 573938 | 120128 | 1475 | Cambisol | Poion alpinae | 10 | 2.93 | 3.15 | 22.06 | 10.75 | 0.1793 |
| **2** | 573993 | 120043 | 1474 | Cambisol | Poion alpinae | 10 | 4.42 | 3.15 | 21.26 | 9.71 | 0.1846 |
| **3** | 573939 | 120045 | 1476 | Cambisol | Poion alpinae | 10 | 2.03 | 2.62 | 12.82 | 7.13 | 0.1815 |
| **4** | 573858 | 119969 | 1492 | Cambisol | Poion alpinae | 10 | 2.12 | 1.36 | 12.95 | 7.16 | 0.1745 |
| **5** | 573849 | 119893 | 1491 | Luvic Cambisol | Poion alpinae | 10 | 1.27 | 2.43 | 14.11 | 8.00 | 0.0142 |
| **6** | 573777 | 119805 | 1506 | Luvic Cambisol | Poion alpinae | 10 | 1.72 | 1.69 | 15.12 | 8.73 | 0.2003 |
| **7** | 573748 | 119746 | 1498 | Cambisol | Poion alpinae | 10 | 1.68 | 2.77 | 14.20 | 7.64 | 0.1588 |
| **8** | 573922 | 119794 | 1485 | Typic Fluvisol | Seslerion | 10 | 1.35 | 1.39 | 18.80 | 9.99 | 0.1649 |
| **9** | 573856 | 119643 | 1500 | Typic Fluvisol | Poion alpinae | 10 | 2.06 | 2.06 | 12.64 | 7.21 | 0.1632 |
| **10** | 574022 | 119804 | 1487 | Cambisol | Rumicion alpini | 10 | 3.84 | 2.42 | 18.47 | 10.10 | 0.1706 |
| **11** | 574020 | 119728 | 1495 | Calcaric Cambisol | Rumicion alpini | 10 | 3.27 | 5.22 | 17.26 | 9.22 | 0.1741 |
| **12** | 574100 | 119724 | 1498 | Calcaric Cambisol | Rumicion alpini | 10 | 4.21 |  | 14.54 | 7.63 | 0.1186 |
| **13** | 574102 | 119642 | 1508 | Calcaric Cambisol | Poion alpinae | 10 | 2.08 | 2.10 | 13.87 | 7.47 | 0.1754 |
| **14** | 574101 | 119563 | 1522 | Calcaric Cambisol | Poion alpinae | 10 | 2.17 | 1.61 | 13.43 | 7.85 | 0.1374 |
| **15** | 573658 | 119476 | 1527 | Cambisol | Poion alpinae | 10 | 1.54 | 1.32 | 14.16 | 7.38 | 0.1155 |
| **16** | 573598 | 119311 | 1545 | Young Fluvisol | Seslerion | 10 | 1.70 | 1.68 | 15.78 | 10.99 | 0.1020 |
| **17** | 573836 | 119563 | 1510 | Typic Fluvisol | Poion alpinae | 10 | 1.80 | 2.33 | 15.64 | 7.86 | 0.1846 |
| **18** | 573840 | 119494 | 1519 | Typic Fluvisol | Poion alpinae | 10 | 2.76 | 1.10 | 16.12 | 7.90 | 0.1313 |
| **19** | 573858 | 119392 | 1536 | Young Fluvisol | Seslerion | 10 | 2.32 | 2.68 | 17.26 | 7.48 | 0.1741 |
| **20** | 573750 | 119318 | 1539 | Young Fluvisol | Seslerion | 10 | 1.71 | 0.84 | 18.64 | 10.01 | 0.0727 |
| **21** | 573757 | 119235 | 1552 | Typic Fluvisol | Seslerion | 10 | 2.11 | 0.92 | 18.34 | 8.79 | 0.1212 |
| **22** | 573840 | 119237 | 1564 | Typic Fluvisol | Seslerion | 10 | 2.03 | 2.27 | 18.80 | 11.01 | 0.1523 |
| **23** | 574166 | 119805 | 1508 | Calcaric Cambisol | Seslerion | 10 | 3.86 | 3.18 | 17.69 | 8.78 | 0.0439 |
| **24** | 574161 | 119753 | 1498 | Calcaric Cambisol | Poion alpinae | 10 | 1.35 | 1.83 | 18.07 | 8.97 | 0.0749 |
| **25** | 573649 | 119380 | 1532 | Young Fluvisol | Poion alpinae | 10 | 1.52 | 1.95 | 17.45 | 10.57 | 0.1540 |
| **26** | 573618 | 119345 | 1539 | Young Fluvisol | Seslerion | 10 | 1.38 | 1.13 | 16.51 | 9.57 | -0.0453 |
| **27** | 573923 | 119727 | 1494 | Typic Fluvisol | Poion alpinae | 10 | 1.53 | 2.47 | 13.61 | 6.97 | 0.1304 |
| **28** | 574004 | 119634 | 1505 | Very young Fluvisol | Petasition paradoxi | 10 | 0.29 | 0.69 | 10.67 | 6.60 | -0.0453 |
| **29** | 573921 | 119555 | 1513 | Typic Fluvisol | Seslerion | 10 | 2.47 | 3.45 | 14.21 | 6.88 | 0.1903 |
| **30** | 574005 | 119559 | 1516 | Very young Fluvisol | Petasition paradoxi | 10 | 0.54 | 0.74 | 10.05 | 6.12 | 0.0924 |
| **31** | 574012 | 119468 | 1525 | Very young Fluvisol | Petasition paradoxi | 10 | 0.50 | 0.76 | 9.39 | 5.08 | 0.0981 |
| **32** | 574013 | 119398 | 1540 | Very young Fluvisol | Petasition paradoxi | 10 | 0.60 | 0.96 | 9.10 | 5.80 | -0.0584 |
| **33** | 574030 | 119312 | 1560 | Very young Fluvisol | Petasition paradoxi | 10 | 1.06 | 0.57 | 8.48 | 4.51 | 0.0496 |
| **34** | 574007 | 119828 | 1480 | Typic Fluvisol | Seslerion | 10 | 1.03 | 1.41 | 18.72 | 11.35 | 0.1204 |
| **35** | 574084 | 119836 | 1495 | Calcaric Cambisol | Poion alpinae | 10 | 1.95 | 3.30 | 17.95 | 10.30 | 0.1706 |
| **36** | 573631 | 119215 | 1551 | Young Fluvisol | Seslerion | 10 | 1.16 | 2.32 | 14.85 | 7.84 | 0.1444 |
| **37** | 573599 | 119150 | 1562 | Young Fluvisol | Seslerion | 10 | 2.70 | 0.67 | 15.16 | 8.55 | 0.1824 |
| **38** | 573604 | 119058 | 1576 | Young Fluvisol | Seslerion | 10 | 1.64 | 1.87 | 13.59 | 9.34 | 0.1330 |
| **39** | 573900 | 119400 | 1537 | Typic Fluvisol | Seslerion | 10 | 1.98 | 2.32 | 17.09 | 7.99 | 0.1785 |
| **40** | 573839 | 119320 | 1549 | Typic Fluvisol | Seslerion | 10 | 1.30 | 1.33 | 16.88 | 8.18 | 0.1745 |
| **41** | 573913 | 119320 | 1557 | Young Fluvisol | Seslerion | 10 | 1.07 | 1.23 | 15.35 | 8.65 | 0.0163 |
| **42** | 573760 | 119152 | 1561 | Young Fluvisol | Seslerion | 10 | 2.62 | 2.71 | 18.75 | 9.22 | 0.0019 |
| **43** | 573834 | 119152 | 1578 | Young Fluvisol | Seslerion | 10 | 1.80 | 2.05 | 19.87 | 9.25 | -0.0129 |
| **44** | 574058 | 119571 | 1516 | Very young Fluvisol | Seslerion | 10 | 0.86 | 1.12 | 13.22 | 7.42 | 0.0885 |
| **45** | 574077 | 119463 | 1530 | Young Fluvisol | Seslerion | 10 | 3.59 | 0.87 | 13.00 | 7.59 | 0.0050 |
| **46** | 574125 | 119480 | 1538 | Young Fluvisol | Seslerion | 10 | 1.30 | 1.02 | 12.71 | 6.90 | 0.0133 |
| **47** | 574014 | 120185 | 1473 | Luvic Cambisol | Poion alpinae | 10 | 1.92 | 1.89 | 18.37 | 9.19 | 0.1243 |
| **48** | 573997 | 120151 | 1473 | Luvic Cambisol | Poion alpinae | 10 | 2.76 | 4.13 | 22.48 | 13.51 | 0.1505 |
