# Supplementary material for: Variation in Soil Respiration across Soil and Vegetation Types in an Alpine Valley
Source: PLoS One. 2016 Sep 29;11(9):e0163968. doi: 10.1371/journal.pone.0163968 (PMC5042455; doi:10.1371/journal.pone.0163968)
Supplement: S3 Table — For each column and flux measurement type, means followed by a different letter are different at the α = 0.05 level. (a) Clipped soil fluxes (Rsc). (b) Vegetated soil fluxes (Rsv). (DOCX) [file pone.0163968.s007.docx]

| (a) Clipped surface | July | August | September | October |
| --- | --- | --- | --- | --- |
| Very Young Fluvisol | 2.0 ± 1.3 a | 2.9 ± 0.7 a | 1.1 ± 0.6 a | 0.5 ± 0.7 a |
| Young Fluvisol | 5.2 ± 0.4 b | 6.6 ± 0.5 b | 2.9 ± 0.3 b | 1.9 ± 0.2 b |
| Typic Fluvisol | 5.2 ± 0.4 b | 7.2 ± 0.5 bc | 3.0 ± 0.3 b | 1.8 ± 0.2 b |
| Calcaric Cambisol | 6.4 ± 0.8 b | 8.3 ± 0.6 c | 4.2 ± 0.4 c | 2.6 ± 0.3 c |
| Cambisol | 7.3 ± 0.6 b | 6.7 ± 0.7 bc | 4.1 ± 0.5 c | 2.6 ± 0.3 c |
| Luvic Cambisol | 6.2 ± 1.1 b | 6.6 ± 0.8 b | 2.8 ± 0.5 b | 2.0 ± 0.4 bc |
| (b) Vegetated surface | July | August | September | October |
| Very Young Fluvisol | 2.4 ± 2.1 a | 5.2 ± 1.0 a | 1.9 ± 0.9 a | 0.8 ± 0.8 a |
| Young Fluvisol | 7.3 ± 0.7 b | 8.4 ± 0.6 b | 3.6 ± 0.4 b | 1.9 ± 0.2 b |
| Typic Fluvisol | 7.1 ± 0.7 b | 9.5 ± 0.7 bc | 3.5 ± 0.4 b | 1.9 ± 0.2 b |
| Calcaric Cambisol | 6.8 ± 1.2 b | 10.5 ± 0.9 c | 5.9 ± 0.6 c | 2.9 ± 0.3 c |
| Cambisol | 7.3 ± 1.0 b | 8.4 ± 0.9 bc | 5.4 ± 0.7 c | 2.6 ± 0.3 c |
| Luvic Cambisol | 8.2 ± 1.7 b | 7.9 ± 1.1 b | 3.5 ± 0.7 b | 2.4 ± 0.4 bc |
