# Supplementary material for: Variation in Soil Respiration across Soil and Vegetation Types in an Alpine Valley
Source: PLoS One. 2016 Sep 29;11(9):e0163968. doi: 10.1371/journal.pone.0163968 (PMC5042455; doi:10.1371/journal.pone.0163968)
Supplement: S4 Table — For each column and flux measurement type, means followed by a different letter are different at the α = 0.05 level. (a) Clipped soil fluxes (Rsc). (b) Vegetated soil fluxes (Rsv). (DOCX) [file pone.0163968.s008.docx]

| (a) Clipped surface | July | August | September | October |
| --- | --- | --- | --- | --- |
| *Petasition paradoxi* | 2.3 ± 2.1 a | 2.5 ± 0.8 a | 0.8 ± 0.5 a | 0.3 ± 1.1 a |
| *Seslerion* | 5.0 ± 0.3 b | 6.8 ± 0.4 b | 2.8 ± 0.1 b | 1.9 ± 0.2 b |
| *Poion alpinae* | 6.1 ± 0.4 b | 7.2 ± 0.4 bc | 3.4 ± 0.2 c | 2.1 ± 0.2 b |
| *Rumicion alpini* | 6.2 ± 0.9 b | 8.4 ± 0.9 c | 6.3 ± 0.4 d | 3.7 ± 0.4 c |
| (b) Vegetated surface | July | August | September | October |
| *Petasition paradoxi* | 3.1 ± 2.9 a | 4.8 ± 1.0 a | 1.4 ± 0.9 a | 0.3 ± 1.2 a |
| *Seslerion* | 7.0 ± 0.5 b | 8.4 ± 0.5 b | 3.4 ± 0.2 b | 1.9 ± 0.2 b |
| *Poion alpinae* | 7.5 ± 0.6 b | 9.2 ± 0.5 bc | 4.1 ± 0.3 c | 2.2 ± 0.2 b |
| *Rumicion alpini* | 6.3 ± 1.3 b | 11.5 ± 1.2 c | 7.7 ± 0.7 d | 3.9 ± 0.5 c |
