# Supplementary material for: Variation in Soil Respiration across Soil and Vegetation Types in an Alpine Valley
Source: PLoS One. 2016 Sep 29;11(9):e0163968. doi: 10.1371/journal.pone.0163968 (PMC5042455; doi:10.1371/journal.pone.0163968)
Supplement: Supplementary file 5 [file pone.0163968.s006.docx]

Plots number	33	30	31	32	28	38	37	46	42	45	44	36	20	43	8	41	16	26	23	21	29	34	19	39	40	22	14	15	17	25	5	18	47	27	48	35	6	24	13	7	2	4	1	9	3	11	12	10
Type of soil	1	1	1	1	1	2	2	2	2	2	1	2	2	2	3	2	2	2	4	3	3	3	3	3	3	3	4	5	3	2	6	3	6	3	6	4	6	4	4	5	5	5	5	3	5	4	4	5
Herbs [%]	55	74	69	74	62	77	94	92	95	72	96	90	94	96	97	97	92	82	96	96	92	96	94	94	98	95	96	99	99	96	97	97	92	99	92	98	99	99	98	96	97	95	97	98	96	92	99	93
Mosses, lichens [%]	4	0.5	15	7.5	22	1	0.5	3	0.5	8	1	2	0.5	0.5	0.5	0.5	1	0.5	0.5	3	0.5	0	0	2.9	0	0.4	0	0	0.4	1	1	0.5	5	0	1	0.5	0	0.5	0	0	0	0	0.5	0.5	0	0	0	0.5
Bare soil [%]	0	0	0	0	0	0	0.5	1	0	0.5	0	0	0	0.5	0	0	1	0.5	0.5	0	1.5	0.1	0	0	0.5	0.1	0.5	0.5	0.5	0.5	0	2	0.5	0	2	0.5	0.5	0.3	1	3	2	2	0	1	2	1	0.5	5
Rocks, pebbles [%]	40	25	15	18	15	15	3	1	0.5	12	1	1	4	0	1	0.5	4	16	3	0.5	0	3.5	0	0.1	0.5	0	3	0.5	0.1	1	2	0	0.5	0	3	0	0	0.2	1	0	0	0	1	0	0	0	0	0.5
Litter [%]	1	0.5	1	0.5	1	7	2	3	4	8	2	7	1.5	3	1.5	2	2	1	0	0.5	6	0.4	6	3	1	4.5	0.5	0.5	0.5	2	0.5	1	2	1	2	1	1	0.5	0	1	1	3	2	0.5	2	7	0.5	1
Species number	24	28	37	39	32	39	40	49	42	45	30	48	43	45	50	33	43	38	42	29	47	41	52	32	35	42	39	45	41	45	50	33	30	31	35	29	37	34	30	26	23	28	18	20	13	28	9	15
Anthyllis vulneraria s.l.	2a	2b	1	2a	2a	+	+	+	1	+	1		1	+	+		2a	r	+			+	+					+			1						1											
Globularia cordifolia	+	1	+	r	1	+		+	+	+			1	r	1		+		+																													
Petasites paradoxus	2b	2a	2b	3	1	2b	3	2b	2a	2a	2b	2a	1	2b	2a	3	1	2b		3	2b	3						1	1	1				2b		2b												
Calamagrostis varia	2a	+	2a	2a	+	1	2a	2a	+	+		1	1	1		+		1								2a			2a	1	1	2a				+												
Hieracium staticifolium			+	1	1																																											
Larix decidua	+	+	1																																													
Gypsophila repens		r	1	+				+																																								
Euphrasia salisburgensis			+	+	1																																											
Tofieldia calyculata	1	1	+	1		+		1	+	+		+		+	+			+																														
Epilobium fleischeri				+	+																																											
Saxifraga aizoides	+			r																																												
Thymus serpyllum aggr.	+	+	+	+	+	+	+	r	+	+	1	+	+	+	1		+		+			1	r	+			+	+			+				1								r					
Campanula rotundifolia		+			+														+											r							r											
Salix appendiculata	+		+		+													+											+																			
Carduus defloratus s.str.		+	+	r	+	1	r					1	+		+			1	+						+						+																	
Dryas octopetala	2b	2a			1	1	1	2a	2a	+		+		+	1																																	
Picea abies			+	+		1								+																																		
Alchemilla conjuncta aggr.		+	+	+	1	+	1	2a	1	2a	2a	+	1	2a	+	+	2a	1	2b	1	2a	+	2a	2a	2a	2a	1	+	+		+			+														
Carex sempervirens	+	1	1	+		2a	1	2a	2b	2a	1	2a	2b	2a	2a	2a	2a		2b	2b	2a	1	2a	2a	2b	2a	1	1	1					+		1												
Lotus corniculatus	+	+	+	1		1	+	1	1	+	r	1	1	1	1	1	1	1	+	+	1	1	+	1	+	+	1	+	+	1	+	+		+		+	+	+	+		+							
Prunella grandiflora	r	2a	1	1		2a	1	2a	1	2a	1	2a	1	2a	1	2a	1	1	2a	2a	+	1	+	+	1	+	+		1	+	+		+	1	+	1		+	1		1							
Carlina acaulis subsp. caulescens	+	2a	1	+	1	1	1	2a	2a	1	2b	1	2a	1	1	2a	1	1	1	+	2a	2a	1	1	1	1	+	1	+	1	1	+		+		1	1	2a										
Galium anisophyllon		+			+		+	+	1	+	+	+	+	+	+	1	+	+	+	+	+	+	+	+	+			r	+		+	+	+		+			+										
Helianthemum nummularium s.l.	r				1	1		1	1	2a		+	1	1	1	1	2a		1	1	2b		2a	+	1	1	+			1	+																	
Phyteuma orbiculare		+		r		+	1	+	+	1	+	+	+	r	r	r	+	r	+	+	+	r	+		r	r	r			+					+		r	+										
Dactylorhiza fuchsii				r		r	1	r		+	+	+	r	r		+		r		r	+		+		r								r															
Carex ferruginea		+		+		+			2a	1	1	1	2a	1	1		1		2b	1	1	2a	2a	1	1	1		1	1	+	+	+		+		1		1	1									
Sesleria caerulea		+		+		1	1	1	2a	2a	1	2a		2a		2a					1		1	2a	2a	2a						+																
Scabiosa lucida	+	+	1	+	r	1	1	+	1	+	+	1	+	+	+		1	+	2a	1	+	r	+	1	+	+		+	r	+	+			+	+	r	+				r							
Polygonum viviparum	r			+	+	+	+	+		+	+	+	+	r	+	+	r	+	+	+	+		+		+	r	+	r		r	+		+		+		+											
Potentilla erecta							r	r	r	+	+	+	1	+	+	+	+		1	+	+	+	1	+	+	1	+	+	+	+	1	+	1		2a		1	+		+	+	+						
Leucanthemum adustum		+	+	+	+	+		+	+	+	+	+	+	+	+	+	+	+	+	+	+	+	+	+	r		+			+						+			+									
Alchemilla vulgaris aggr.															1				+	1	+	1	1	2a	2a	2b	2a	2a	1	2b	1	2a	2a	2a	2b	+	2a	2b	2a	2b	2a	+	2b	2a	2a	2b	+	
Deschampsia cespitosa														1	1			1							+	+		1	1	1	1	+	2a	1	2a	2b	1	1	+	1	2a	2a	1	2a	2a	+		+
Trollius europaeus													+	+		+			+	1	r	1	2a	1	1	1	1	2a	2b	2a	1	2b	1	1	+	+	1		1	1	1	1		1	1			
Ranunculus montanus				+			r	+	+		+	+	+	+	+	+	+	+		1	+	+	+	+	1	1	2a	+	2a	1	1	1	2a	+	2a	1	1	1	1	2a			2a	+	2a	1		
Trifolium pratense			+		+	+	+	+	1	+	1	1	+	2a	1	1	2a	1	+	1	2a	1	1	2a	1	1	2a	1	1	1	2a	2a	1	1	1	2a	1	1	1	2a	1	1	1	1		1		
Dactylis glomerata					1	+	+	+	1				1	1	1		1	1	+		r	1	r			1	+		2a	1	1	2a		2b	1	+		+	1	+	2b	2b		2b	3			
Plantago media											r		1		1						+	+	+			+	2b	1		+	2a				1	1	1	1		1	+	1	2a	1				r
Cynosurus cristatus																															+	+		+			+		1	+								
Ranunculus repens																																							+	r						1	1	1
Rumex alpinus																																														2a	4	1
Festuca pratensis s.str.																													+																	1		1
Plantago major s.str.													1		1							1			+		1		+	+	1				+		1		1	+	1		1		+	1	1	1
Trifolium repens s.str.																		1	+		+	+		+		1	1	1	1	1	1	2a	1	1	+	2a	1	1	1	2a	2a	1	1	2a	1	+	2a	2b
Bellis perennis																																							+							+		+
Poa supina																																	1		1											2a		3
Veronica serpyllifolia s.l.																																														+		+
Chaerophyllum hirsutum																																						+				1				+		r
Carum carvi															1			+			+	+					1		1	2a	+	+		2a		1		1	1	1	1	1	1	1	1	2a	r	1
Taraxacum officinale aggr.			+												1							+				+		1	+	1	1			1		2b		2a	1	1			1	2b	1	2a	+	+

Plots number	33	30	31	32	28	38	37	46	42	45	44	36	20	43	8	41	16	26	23	21	29	34	19	39	40	22	14	15	17	25	5	18	47	27	48	35	6	24	13	7	2	4	1	9	3	11	12	10
Festuca rubra aggr.				+	+	1	+	1	+	1		1	+		1	+	1	1	+	+	2a	1	2a	2a	+	1	+	1		1	1		1	1	2a	+	1	+	+	1	1		1	1		1		
Euphorbia cyparissias	+	+	+	+			+	+	+	+	+	1	1	1		1	+		1	1	r	+	1	1	1	1	1	+	+	1		1			r	r	1					+		+				
Agrostis stolonifera aggr.			+	+	+		+	+			+	+		+			+	+				+		+	1	+	+	+	+	+	+	+	+	+	+	+	+	+			+	+		+	+			
Briza media		+			r			+	r	+		+	+	+	+	+	+	+	+	+	r	+			+		+	r			+			r	+		+											
Rhinanthus alectorolophus			1	1	r	r		+		+		+		1			r		+	1	1	+	+	2a		1	+	+	r	+				1				1										
Carex sylvatica			+									+					1	+						+	+	1	+	1		+	1		1		1	+	1		2a	2b		2a	1	+		1		
Soldanella alpina							+	+	+	+				+	+				r		+		+	+	+			+	+	r	+	+	1					+	+			+	+					
Euphrasia hirtella	+		+	r		1	+	+		+		+	+		+	+	+		+	+		+					+	+								+		+	+									
Hieracium villosum			r		+		+			+	+				+			1								+		2a		1	1			+			1	+	1	1		1	+					
Veronica chamaedrys												r									+		+	r		+			+	+		1	+	+	+		+	+		r		+		+		+		
Luzula sieberi									+			+	+	+	+	+	+		+	+			+	1	+	1				+	+	1	1															
Ranunculus acris subsp. friesianus							+							1	1				+	1	+		+			+								1				+	+		2a	2a		1	1	2a		+
Parnassia palustris	+		+	r		+	+	+	+					r				r	+			+					+	+		+						r		+										
Astrantia major													r			r	r	r	1				1	+		+		1	+	1						r	+			+		1						
Pimpinella major										+		+					+					1				+		+	r		+	+	+		1		+		r			+						
Gentiana lutea					+	r	1					1	+				1			r							1	2a		1	1				1					+		1						
Leontodon autumnalis											1							+							+	+	1	+	r		+			+					2a				2b		+	+		
Onobrychis montana		+				+	+	1	+	1	+	+			1		1		1								+				1																	
Hippocrepis comosa					1	1	r		+	1	r	+	+	+			+				+		+								+																	
Carex ornithopoda	+		+	+				+		1			1	+	+						+		+				1		+						1													
Aster bellidiastrum			+	1		+		+		+		r							+	+	+		+	+									r								r							
Plantago lanceolata												+									+					+	+	+						+			+	2a			+	+	+	1				
Gentiana asclepiadea						+					1	1			1			1		1	+	1	+						1	+		1																
Hieracium bifidum aggr.				+	r			+	+	+			+	1				+			+		+			+									1													
Plantago atrata s.str.								+									+					1	+			1		+		+	+			1						+			2a			1		
Carex flacca										+				+				1	1		1		+				2a	2a			+							1	2b									
Cirsium acaule									1	+			1		1	1					+	+	+			1	2a			1																		
Ajuga reptans									r			r		+				+					+			+		r	+				r							+			+					
Linum catharticum	r							r				+	r		+		r											r		r	+						r							r				
Poa alpina										r	+										1	+					1		+	+		+	r	+												1		
Thesium alpinum			r	+		+		+	+	r	+	+															+				+						+											
Aposeris foetida																+					+	+	+	1	1	2a		+	+				2a															
Cerastium fontanum subsp. vulgare			+																			r	+		+				r			r						r	+			+				+		
Leontodon hispidus s.str.		+	1	r											2a							1	+	+					1			+				1												
Selaginella selaginoides	+	+				+	+	+	+	+	+		+		+																																	
Polygala chamaebuxus		+					+		1	2a		+			+				+												+						+											
Pimpinella saxifraga					+								+		+	+						r		+										+		1										r		
Polygala alpestris	+		r	r		+	+	+		+											r																	+										
Silene vulgaris s.l.												r	r	r		+		+	r													r		r														
Campanula scheuchzeri			+		r											+									r	r							r								+	+						
Prunella vulgaris															+												+				2a	1					2b		1					+				
Crepis aurea					r													1										2a		+	1						+				1							
Chaerophyllum villarsii												+					+											+		+	+						+			+								
Gymnadenia conopsea						+		+	+							+	r				+		r																									
Hypericum maculatum s.str.																							r						+			+									1	1		r				
Campanula rhomboidalis																			+									+			+		+					r		+								
Trisetum flavescens																											+				2a						1	+	1							+		
Hieracium pilosum								+	r						r		r		r																+													
Viola biflora																		+					+					+					+					+										
Gentiana campestris s.str.									+						+		+								r												1											
Phleum hirsutum								+					+								r				+							1																
Phleum rhaeticum																	r																		+					+		+				+		
Geranium sylvaticum																							1					+	2a			1															1	
Rumex alpestris																																	r								+	1			+			
Fragaria vesca							+						r			+	+																															
Anthoxanthum odoratum s.l.								+									1				+		+																									
Crepis pyrenaica																+								+						+					r													
Alchemilla glabra aggr.																														+									1			2b						2a
Acer pseudoplatanus				+		+	r										r																															
